# Accounting for trait architecture in genomic predictions of US Holstein cattle using a weighted realized relationship matrix

**DOI:** 10.1186/s12711-015-0100-1

**Published:** 2015-04-02

**Authors:** Francesco Tiezzi, Christian Maltecca

**Affiliations:** Department of Animal Science, North Carolina State University, Raleigh, NC 27695 USA

## Abstract

**Background:**

Genomic BLUP (GBLUP) can predict breeding values for non-phenotyped individuals based on the identity-by-state genomic relationship matrix (**G**). The **G** matrix can be constructed from thousands of markers spread across the genome. The strongest assumption of **G** and consequently of GBLUP is that all markers contribute equally to the genetic variance of a trait. This assumption is violated for traits that are controlled by a small number of quantitative trait loci (QTL) or individual QTL with large effects. In this paper, we investigate the performance of using a weighted genomic relationship matrix (**wG**) that takes into consideration the genetic architecture of the trait in order to improve predictive ability for a wide range of traits. Multiple methods were used to calculate weights for several economically relevant traits in US Holstein dairy cattle. Predictive performance was tested by k-means cross-validation.

**Results:**

Relaxing the GBLUP assumption of equal marker contribution by increasing the weight that is given to a specific marker in the construction of the trait-specific **G** resulted in increased predictive performance. The increase was strongest for traits that are controlled by a small number of QTL (e.g. fat and protein percentage). Furthermore, bias in prediction estimates was reduced compared to that resulting from the use of regular **G**. Even for traits with low heritability and lower general predictive performance (e.g. calving ease traits), weighted **G** still yielded a gain in accuracy.

**Conclusions:**

Genomic relationship matrices weighted by marker realized variance yielded more accurate and less biased predictions for traits regulated by few QTL. Genome-wide association analyses were used to derive marker weights for creating weighted genomic relationship matrices. However, this can be cumbersome and prone to low stability over generations because of erosion of linkage disequilibrium between markers and QTL. Future studies may include other sources of information, such as functional annotation and gene networks, to better exploit the genetic architecture of traits and produce more stable predictions.

**Electronic supplementary material:**

The online version of this article (doi:10.1186/s12711-015-0100-1) contains supplementary material, which is available to authorized users.

## Background

Since the introduction of dense single nucleotide polymorphisms (SNP) chips, the statistical methodology used in genomic selection has undergone significant improvements. Prediction of genetic merit is most commonly based on linear regression models, in which the genomic breeding value (GEBV) for an individual is computed as the sum of the marker effects multiplied by the specific individual allelic variants across the entire genome [1]. Since the number of markers that are simultaneously fitted in the model can be high, different strategies that deal with small *n* and large *p* (where *n* is the number of genotyped individuals and *p* is the number of predictors) have been adopted. Each method leans more or less on a priori assumptions about the genetic architecture of the trait and the linkage disequilibrium (LD) between markers and the quantitative trait loci (QTL) [2].

Alternatively, genomic-BLUP (GBLUP) can take advantage of the genomic relationship matrix (**G**) constructed from genotypic information [3-5], where additive genetic relationships between pairs of individuals are derived from the number of alleles shared at each locus of the genome. GBLUP assumes a polygenic architecture of the trait analyzed and considers the contribution of all the markers in the construction of the **G** matrix. This is opposed to other methods such as those of the Bayesian alphabet family, which often assume that the genetic variance is explained by a reduced number of markers, among which some have a small or no effect [6-8]. Based on this assumption, performance of GBLUP (and Bayesian Ridge-Regression (RR)) is expected to be poorer than other methods when the trait is not polygenic. This performance decline occurs when for example the number of QTL is smaller than the number of effective independent loci across the genome. In a simulation study, Daetwyler et al. [2] showed a clear advantage of Bayes-B over GBLUP for traits for which the number of QTL was small in comparison to the significantly smaller number of independent chromosomal segments, while this advantage was lost for highly polygenic traits.

Since GBLUP measures the average relationship among individuals across the genome, it is less sensitive to the genetic architecture of any particular trait. Thus, GBLUP predictions lean more on within-family linkage and Mendelian sampling, while LD between markers and QTL becomes of secondary importance. Habier et al. [6,9] demonstrated how the decaying genetic relationship across generations affected RR prediction accuracy more than that obtained with Bayes-B because RR is more sensitive to family linkage. This is mainly because it forces all markers to contribute to genetic variation and neglects the fact that only some markers may be in LD with the QTL. Conversely, GBLUP presents some advantages in the implementation of various models. All extensions of BLUP, such as multiple-trait, random regression, probit and logit models can be easily accommodated and made computationally efficient. Moreover, the GBLUP method has been extended to the single-step BLUP method, which allows the incorporation of both pedigree-derived and genomic-derived relationships into a single relationship matrix [10].

The assumption of polygenic architecture represents a potential disadvantage of GBLUP since the model does not explicitly allow regions near QTL to absorb more genetic variation than other regions. Nevertheless, contributions from each marker to **G** can be manipulated in the computational process, such that the assumption of equal contribution can be relaxed. In the basic **G** matrix [11], markers are weighted by their expected variance, i.e. weights become solely a function of allele frequencies. Alternatively, the relative emphasis of each marker can be adjusted by the real contribution of the specific locus to the total genetic variation of the trait, such that individuals will be more related if they share the same copy of a locus with a large effect, rather than other loci. Studies have shown that more emphasis can be given to markers with larger effects on the trait of interest, resulting in an increase in predictive ability of the model [12,13] and reduction in bias [14,15]. For instance, marker information can be obtained from prior genome-wide association studies for the trait of interest. It is clear that larger gains are expected for traits with few QTL [12], since the underlying **G** matrix assumptions are violated to a greater extent.

The use of a weighted **G** matrix has been tested [12-15] mainly in simulated data, although examples exist for human [7] and livestock data [16] as well. However, effectiveness and consequences of the weighting procedure have not been investigated on a large series of economically relevant traits with different genetic architectures [17]. The aim of this study was to test the predictive ability of different BLUP equations using a weighted **G** matrix across nine traits of varying genetic architecture and heritability in US Holstein dairy cattle.

In the current study, three different weighting methods were compared to the traditional genomic matrix and pedigree-derived matrix. Performances were measured in terms of accuracy and bias of prediction in a cross-validation scheme.

## Methods

### Data and genotypes

Genomic and phenotypic data were obtained from the Bovine Functional Genomics Laboratory and Animal Improvement Programs Laboratory at the USDA-ARS Beltsville Agricultural Research Center (Beltsville, MD). De-regressed measures were derived from sires’ predicted transmitting ability values (PTA) from US national genetic evaluations for production, calving ease and type traits and used as pseudo-observations for subsequent analyses, given that these provide some advantages compared to other sources [18].

The first group of traits analyzed included milk yield (MY), fat percentage (FP), and protein percentage (PP); the second group of traits included direct calving ease (DC) and maternal calving ease (MC); the third group of traits included body depth (BD), rump width (RW), stature (ST) and strength (SR). Heritabilities were obtained from VanRaden et al. [17] and are reported in Table 1. The traits were chosen to represent a large range of heritabilities and genetic architectures, ranging from high heritability and low number of QTL (e.g. FP) to low heritability and QTL with large effects (e.g. DC) and low heritability and high number of QTL (e.g. MC).

**Table 1:** **Descriptive statistics** ^**1**^ **for the PTA dataset used, for each medoid and for the whole dataset**

**Trait** ^**2**^	**h** ^**2**^	**Medoid 1 n = 1004**	**Medoid 2 n = 1069**	**Medoid 3 n = 1168**	**Medoid 4 n = 1104**	**All n = 4865**
MY	0.30	712.9^(0.896)^	584.4^(0.897)^	556.3^(0.902)^	544.3^(0.887)^	592.2^(0.896)^
FP	0.50	0.893^(0.896)^	−1.837^(0.897)^	1.197^(0.902)^	0.606^(0.887)^	−0.014^(0.896)^
PP	0.50	1.161^(0.896)^	0.733^(0.897)^	0.979^(0.902)^	1.109^(0.887)^	1.185^(0.896)^
DC	0.09	7.493^(0.806)^	8.046^(0.794)^	8.141^(0.793)^	7.933^(0.782)^	7.938^(0.793)^
MC	0.06	8.622^(0.742)^	8.823^(0.731)^	8.610^(0.731)^	8.708^(0.716)^	8.682^(0.730)^
BD	0.37	−0.129^(0.883)^	−0.173^(0.880)^	−0.029^(0.881)^	0.079^(0.874)^	−0.057^(0.879)^
RW	0.26	−0.451^(0.882)^	−0.020^(0.881)^	−0.054^(0.881)^	−0.006^(0.877)^	−0.118^(0.880)^
ST	0.42	−0.101^(0.887)^	−0.096^(0.887)^	−0.205^(0.890)^	0.223^(0.887)^	−0.062^(0.888)^
SR	0.31	−0.207^(0.882)^	−0.066^(0.880)^	−0.047^(0.880)^	0.001^(0.876)^	−0.073^(0.880)^

^1^Descriptive statistics are the mean and standard deviation; ^2^traits were milk yield (MY), fat percentage (FP), protein percentage (PP), direct calving ease (DC), maternal calving ease (MC), body depth (BD), rump width (RW), stature (ST), and strength (SR).

PTA were de-regressed prior to analysis following Garrick et al. [18]. Sires were required to have a reliability of de-regressed PTA (dePTA) higher than 0.2 for all traits to enter subsequent analyses. Genotypes were from the 50 K Illumina Beadchip. Standard editing criteria included the removal of SNPs with a minor allele frequency less than 0.05 and a call rate less than 0.99. Markers that were unmapped or mapped to the sex chromosomes were removed. Finally, individuals with a call rate less than 0.99 were discarded. The remaining missing SNPs were imputed using Beagle [19]. After editing, genotypes on 39 004 SNPs for 4865 US Holstein bulls were available.

### Marker selection methods

Weighting the **G** matrix requires an estimate of marker effects [12,14,15]. In the present study, marker solutions were obtained according to three different methods that were chosen on the basis of their statistical properties, treatment of the marker effect and correction for population stratification. All methods considered an additive polygenic effect in the model to account for population structure.

The first approach used for weighting was based on feature selection through single-marker regression. Only significant SNPs (p < 0.05) were used to construct the **wG** matrix, as already proposed by de los Campos et al. [7]. The single-marker regression (SM) model was as follows:

$$ {y}_{ij} = {x}_i{b}^m+{z}_i{s}_j+\frac{e_{ij}}{w_{ij}}, $$

where *y*_*ij*_ is the pseudo-phenotype for *i*^*th*^ individual sired by the *j*^*th*^ sire, *b*^*m*^ are the mean and *m*^*th*^ marker fixed effect, *s*_*j*_ is sire additive genetic effect, where **s** ~ *N*(0, **A**σ^2^_s_), with **A** representing the additive relationship matrix derived from the pedigree, *e*_*ijk*_ is the residual, and *w*_*ijk*_ is the weight of the *y*_*ijk*_ dePTA, as proposed in Garrick et al. [18], *x*_*i*_ is the *i*^*th*^ row of the **X** incidence matrix reporting a vector of 1 s and the number of copies of the minor allele (0, 1 or 2) for the *i*^*th*^ individual, and *z*_*i*_ is the *i*^*th*^ row of the **Z** is the incidence matrix for the sire effect. The significance (*P-value*) of the F-test for the marker effect was used to select SNPs. Analyses were performed in R using the package ‘pedigreemm’ [20].

The other two methods involved fitting all markers simultaneously as random effects, modeled in a Bayesian framework [21]. These methods were chosen to obtain values of the expected variance that is absorbed by each marker to be used in weighting the genomic matrix, instead of selecting only the SNPs with the strongest impact. The general model was:

$$ {y}_i = {1}^{\hbox{'}}\mu +{\sum}_{j=1}^m{w}_{ij}{u}_j+{z}_i{a}_i+\frac{e_i}{w_i}, $$

where *μ* is the mean of the population, *u*_*j*_ is effect of the *j*^*th*^ marker, *a*_*i*_ is the additive polygenic effect of the *i*^*th*^ individual assuming the vector **a** ~ *N*(0, **A**σ^2^_s_), with **A** representing the additive relationship matrix derived from the pedigree, *e*_*i*_ is the residual, *w*_*i*_ is the weight of the *y*_*i*_ dePTA, *w*_*ij*_ is the genotype of sire *i* at marker *j*, and *z*_*i*_ is the *i*^*th*^ row of the additive polygenic effect incidence matrix **Z**.

Two approaches were used to estimate marker effects: Bayesian Ridge-Regression (RR) and Bayesian LASSO (BL). These were chosen because they differ in the prior assumption on marker effects and their variances, as well as the penalization criteria applied to the estimation of these parameters [22,23]. RR was chosen because it assumes a completely polygenic architecture. Likewise, BL was chosen because it applies a non-fixed shrinkage of marker effects, their intensity being inferred from the data. This makes this method more sensitive to the genetic architecture of the trait. All analyses were performed in R using the package ‘BLR’ [24].

Prior specifications used here were defined following de los Campos et al. [24]. For all models, priors for additive polygenic genetic effects *a* were multivariate normal $\mathbf{a}\sim N\left(0,\mathbf{A}{\sigma}_a^2\right)$, where **A** is the pedigree-derived numerator relationship matrix, and priors for residual (${\sigma}_e^2$) and additive polygenic variance $\left({\sigma}_a^2\right)$ followed an inverted chi-squared distribution *inv-χ*^*2*^*(ν, S)*, where ν represents the degrees of freedom and *S* is the scale. In RR, the prior for marker effects was $\mathbf{u}\sim N\left(0,\mathbf{I}{\sigma}_u^2\right)$, where ${\sigma}_u^2$ is the marker variance that follows an inverted chi-squared distribution inv-χ^2^(ν, S). While ν was arbitrarily equal to 5 for all models, *S* was chosen according to the expectation of variance for the specific trait. Therefore, given V_y,_ the variance for the pseudo-phenotypes, and ${V}_y\left(\frac{1}{2}{h}^2\right)$ as expectation of additive polygenic and total genomic variance, additive polygenic effect variance had a scale ${S}_a = {V}_y\left(\frac{1}{2}{h}^2\right)\left(\nu -2\right)$ and residual variance had a scale *S*_*e*_ = *V*_*y*_(1 − *h*^2^)(*ν* − 2). Priors for the total genomic variance require that the variability of SNP allele frequency is known, which can be summarized as $M{S}_w={n}^{-1}{\sum}_{i=1}^n{\sum}_{j=1}^m{\left({x}_{ij}-{\overline{x}}_j\right)}^2$, where *n* is the number of individuals, *m* is the number of markers, *x*_*ij*_ is the number of copies of the minor allele, and *x*_*j*_ is the average for marker *j*. According to this, the scale for genomic variance in RR was ${S}_{RR} = \left[{V}_y\left(\frac{1}{2}{h}^2\right)\left(\nu -2\right)\right]/M{S}_w$. In BL, marker effects were assumed to follow a double-exponential distribution, with a parameter τ^2^ regulating the amount of shrinkage. This value τ^2^ followed an exponential distribution Exp(τ^2^|λ), where λ is a regularization parameter for the shrinkage of marker effect estimates. In this study, λ was considered random and assigned a gamma distribution G(λ^2^|α_1_,α_2_), with given shape α_1_ and rate α_2_. While α_1_ was set equal to 1.01, we derived ${\alpha}_2=\left({\alpha}_1-1\right)\left(\frac{h^2}{2\left(1-{h}^2\right)M{S}_w}\right)$, so that we obtain the expectation of the regularization parameter $\lambda =\sqrt{2\frac{\left(1-{h}^2\right)}{h^2}M{S}_w}$, which was specific for each trait. The population mean was sampled from a flat prior. Both RR and BL chains were run for 120 000 iterations, with 20 000 iterations as burn-in and thinning every 10 iterations. Convergence was assessed by visual inspection of trace plots and running postgibbs analyses using the ‘coda’ R package [25].

### Genomic relationship matrices

For all genomic relationship matrices, we partially rearranged the procedure reported in [11]. Marker incidence matrix **W** (with entries reporting the number of copies of the minor allele as 0, 1 or 2) was converted into **M** by subtracting 1, such that entries were −1, 0 and 1. Then, a vector **t** of length *m* was computed such that the entry for the i^th^ marker was t_i_ = 2(p_i_-0.5), where p_i_ is the minor allele frequency. Matrix **Z** was obtained by subtracting **t** from **Z**. For ease of computation, instead of building **G** = **ZZ’** directly, we constructed 39 004 single-locus genomic relationship matrices. For each z_i_ column of **Z** we computed **G**_**i**_ = z_i_z_i_’. The genomic relationship matrices were computed as a weighted sum of the single-marker genomic matrices for each of the methods, corrected for mean identity by state (IBS) relatedness. This method of constructing genomic matrices as a weighted average of several single-marker genomic matrices has already been proposed and used by Zhang et al. [12]. We defined different weights for the different matrices. Given p_i_ as the minor allele frequency and w_i_ as weight for the i^th^ marker, the following relationship matrices were built.

In **G**^**BASE**^ the weight was calculated as:

$$ {w}_i=2{p}_i\left(1-{p}_i\right), $$

The weighting applied to the base genomic matrix is a modification of the second method described by VanRaden [11], where markers contribute to genomic relatedness proportional to the reciprocal of their expected variance. In this study, the weight assigned to each marker is the expected variance.

When information on marker contribution was provided from different sources (SM, RR, BL), it was possible to weight the contribution to the genomic matrix by the marker-specific realized variance. In the SM, the weight on marker *i* in the weighted **G** matrix (**G**^**SM**^) was:

$$ {w}_i=2{p}_i\left(1-{p}_i\right){S}_i, $$

where S_i_ is an indicator with value ‘1’ assigned to markers for which P-value < 0.05, and ‘0’ otherwise.

Finally, from the two Bayesian approaches RR and BL, matrices **G**^**B**L^ for **G**^**RR**^ were constructed with weights:

$$ {w}_i=2{p}_i\left(1-{p}_i\right){\hat{u}}_i^2, $$

where ${\hat{u}}_i$ is the allele substitution effect for the *i*^th^ marker.

### Genomic BLUP

For all traits, predictions were calculated using GBLUP, using different genomic matrices (**G**^**BASE**^, **G**^**SM**^, **G**^**RR**^, **G**^**BL**^) in turn. In addition, a prediction from a pedigree-derived relationship matrix (**A**^**PED**^) was obtained in order to facilitate comparisons. MME were:

$$ \left[\begin{array}{cc}\hfill \mathbf{1}\mathbf{\hbox{'}}\mathbf{1}\hfill & \hfill \mathbf{1}\mathbf{\hbox{'}}\mathbf{Z}\hfill \\ {}\hfill \mathbf{Z}\hbox{'}\mathbf{1}\hfill & \hfill {\mathbf{Z}}^{\hbox{'}}\mathbf{Z}+{\mathbf{G}}^{-1}\uplambda \hfill \end{array}\right]\left[\begin{array}{c}\hfill \hat{\boldsymbol{\upmu}}\hfill \\ {}\hfill \hat{\mathbf{u}}\hfill \end{array}\right]=\left[\begin{array}{c}\hfill \mathbf{1}\mathbf{\hbox{'}}\mathbf{y}\hfill \\ {}\hfill \mathbf{Z}\hbox{'}\mathbf{y}\hfill \end{array}\right], $$

where **1** is a vector of length *n* containing values of ‘1’, **Z** is a *n* by *n* incidence matrix for the additive genetic effect of the individual, **G** is the *n* by *n* relationship matrix to be tested, **y** is a vector of size *n* containing the weighted dePTA (the product of the dePTA and its weight, as described previously), $\hat{\mu}$ is the solution for the population mean, $\hat{u}$ is the solution for the additive genetic effect, $\lambda =\frac{\left(1-{h}^2\right)}{h^2}$, and h^2^ is the assumed heritability. Equations were solved by direct inversion and the vector of predicted values was obtained as $\hat{\boldsymbol{y}}={\mathbf{1}}^{\boldsymbol{\hbox{'}}}\hat{\boldsymbol{\mu}}+{\boldsymbol{Z}}^{\boldsymbol{\hbox{'}}}\hat{\boldsymbol{u}}$. In order to assess the impact of the shrinkage parameter on the predictive ability of the different relationship matrices, several values of λ were tested for heritabilities ranging from 0.1 to 0.9, in 0.1 step increases. This parameter did not affect the predictive ability of the different models. However, for each model we considered the predictive ability of the value of λ that gave the best fit: given **y** as the phenotype and **ŷ** as the vector of predicted values for the *n* masked observations in cross-validation, we used the value of λ that gave the solutions that minimized the difference $d={\sum}_{i=1}^n{\mathrm{y}}_{\mathbf{i}}-{\widehat{y}}_{\mathbf{i}}$.

### K-means cross-validation

To test the predictive ability of the models, a 4-fold k-means cross-validation was performed [26]. Dissimilarities between individuals were derived from **A**, and were used as sources of information to separate individuals across medoids, i.e. clusters of individuals that were formed to maximize intra-group and minimize inter-group additive genetic relationships. Relative distances were computed as:

$$ {d}_{ij}=1-\frac{a_{ij}}{\sqrt{a_{ii}*{a}_{jj}}}, $$

where *d*_*ij*_ is a measure of pedigree distance between individuals *i* and *j*, *a*_*ij*_ is the additive genetic relationship between the two individuals, *a*_*ii*_ and *a*_*jj*_ are the diagonal elements of the relationship matrix for individuals *i* and *j*, respectively. Each medoid was used as a validation set using the remaining three as the training set. Predictions (**ŷ**) from each model were regressed on the weighted dePTA (**y**) to obtain measures of accuracy and bias. The former was assessed as the correlation between **y** and **ŷ**, while bias was measured as the regression coefficient from the linear model $\boldsymbol{y}=\boldsymbol{a}+\boldsymbol{b}\hat{\boldsymbol{y}}+\boldsymbol{e}$. Because true breeding values were not available, correlation coefficients were weighted by the average PTA reliability of the medoid [9,27], as reported in Table 1. Accuracy and bias were averaged across the four folds used in cross-validation in order to have a single measure per model. Relative gain in accuracy was computed for each model as the difference between the accuracy of that model (**G**^**SM**^, **G**^**RR**^, or **G**^**BL**^) and **G**^**BASE**^, divided by the accuracy of **G**^**BASE**^.

### Accounting for population stratification

One of the major concerns of all weighting procedures stems from the difficulty to separate population stratification from actual QTL signals [28]. In genomic predictions, an additive polygenic effect is often considered in the model for the purpose of correcting for population stratification [29]. In this work, we considered an additive polygenic effect in the estimation of marker effects but it may not completely correct for stratification. If population stratification and family linkage are not appropriately accounted for, they may lead to biased estimation of marker effects, which in turn may lead to overestimation of prediction accuracy.

We used two different empirical ways to assess the impact of spurious associations.

First, results from genome-wide regression analysis were cross-referenced for consistency with associations reported in the literature. This was done in order to verify the validity of the associations obtained by the different methods. The full list of QTL for each of the traits analyzed was extracted from the Animal Genome QTL database [30] and mapped to chromosomes 1 to 29. One Mb sliding windows were then created, and were assigned a value of ‘1’ if they contained at least one QTL (for the specific trait), and ‘0’ otherwise. From marker effects calculated in the present study, the variance accxounted for by the sliding windows was computed as:

$$ {V}_n = {\sum}_{i=\mathrm{a}}^b2{p}_i\left(1-{p}_i\right){\hat{u}}_i^2, $$

where *V*_*n*_ is the variance for the *n*^*th*^ window, including markers from the start position *a* to the stop position *b*, *p*_*i*_ is the MAF for the *i*^*th*^ marker and ${\hat{u}}_i$ is the average allele substitution effect for the *i*^*th*^ marker. In this case, the marker effects were derived using RR since this method allows fitting all markers simultaneously in the model (i.e. taking LD into account), with the same penalization across the genome (i.e. no marker-specific shrinkage). The values of ${\hat{u}}_i$ used here were averages of the marker effects obtained across the four training replicates in the cross-validation. For each trait, windows were ranked for descending values of cumulative absorbed variance and the top *t* windows (with values of *t* = 0.010%, 0.025%, 0.050%, 0.100%, 0.250%, 0.500%, 1.00%, 2.5%, 5%, 10%, 25% and 50%), were in turn declared significant and assigned a value of ‘1’, while the other non-significant windows were assigned a value of ‘0’. Once the different sets of significant markers were defined, the sensitivity to marker effect inflation was measured using false positives rate (FPR) computed as the proportion of windows that were declared significant but that did not contain a reported QTL over the total number of windows declared significant. This analysis is sensitive to the number of QTL reported in the database, therefore FPR was reported only for production traits, for which a large number of reported QTL are available. It should be noted that these three traits (MY, FP, and PP) are not completely representative of the genetic architecture of all traits under selection in dairy cattle but offer a framework for the interpretation of the overall results.

Furthermore, we attempted to verify the possibility that family structure was still present in the weighted genomic matrices. In the pedigree-derived matrix **A**, relationships among individuals are the expected identical by descent (IBD) probabilities under the Fisher model and depend essentially on the completeness of the pedigree [4]. Conversely, the genomic relationship matrix **G** relates individuals based on the number of alleles they share at each locus, regardless of ancestry (i.e. IBS). The weighted genomic relationship matrix **wG** is in turn expected to relate individuals based on the shared number of copies of a given allele at a QTL (or at the loci in linkage with the QTL, if this is unknown).

The amount of inflation in prediction accuracy due to population stratification and still present in the weighted relationship matrix can be assessed empirically as the relative distance between the three matrices **A**, **G**^**BASE**^, and **wG**. We speculate that if an association with a QTL was not found due to lack of statistical power, **wG** will be close to **G**^**BASE**^ and both **wG** and **G**^**BASE**^ will be roughly equidistant from **A**. A similar situation will arise by lack of association due the polygenic architecture of the trait. Conversely, if **wG** is still tracing population or family structure, its distance from **G**^**BASE**^ will increase, while the distance from **A** should decrease, under the assumption that **wG** re-traces expected relationships. Finally, if the weighting procedure correctly pinpoints significant regions of the genome, **wG** should depart from **G**^**BASE**^, as well as from **A**. It should be noted that, within this framework, the lack of statistical power and the genetic architecture are not a concern since, in these cases, **wG** should essentially be equal to **G**^**BASE**^ and the two procedures should produce the same results.

A measure of relative distance between matrices was obtained that assigned individuals to different groups. A specific **wG** was built for each trait using the formulas reported above. In this case, and unlike in the cross-validation analysis, marker effects used were from RR estimates averaged over the four replicates. For each trait, the k-means clustering was repeated over the three matrices and individuals were assigned to the four different groups based on the genetic distance used to obtain the medoids. For each matrix, individual pair-wise comparisons were assigned the value ‘1’ to bins that appeared in the same medoid (regardless of the medoid label) and ‘0’ otherwise. The proportion of bins of **wG** that fell in the same medoid in comparison with either **G**^**BASE**^ or **A** over the total number of pairs was then measured. This yielded an empirical relative distance between the three matrices for each trait.

## Results

### Obtaining marker weights and accounting for population stratification

PTA used in the analyses and their reliabilities are summarized in Table 1 for each medoid and for the total dataset. PTA are expressed on the scale used for US national genetic evaluations [17]. In general, production traits had the highest reliabilities (0.896 for the whole dataset), followed by type traits (from 0.879 to 0.888), while calving ease traits had the lowest reliabilities (from 0.730 to 0.793). The number of medoids to perform k-means clustering was set to four based on a preliminary analysis (results not shown). The number of bulls was equal to 1004, 1069, 1168 and 1104 for medoids M1 to M4, respectively, as reported in Table 1. The four medoids, as separated by k-means, represented groups that varied both in PTA and reliabilities. The highest range in reliability across the medoids was found for MC, although the difference was still negligible (0.742 for M1 and 0.716 for M4).

**Figure 1:**
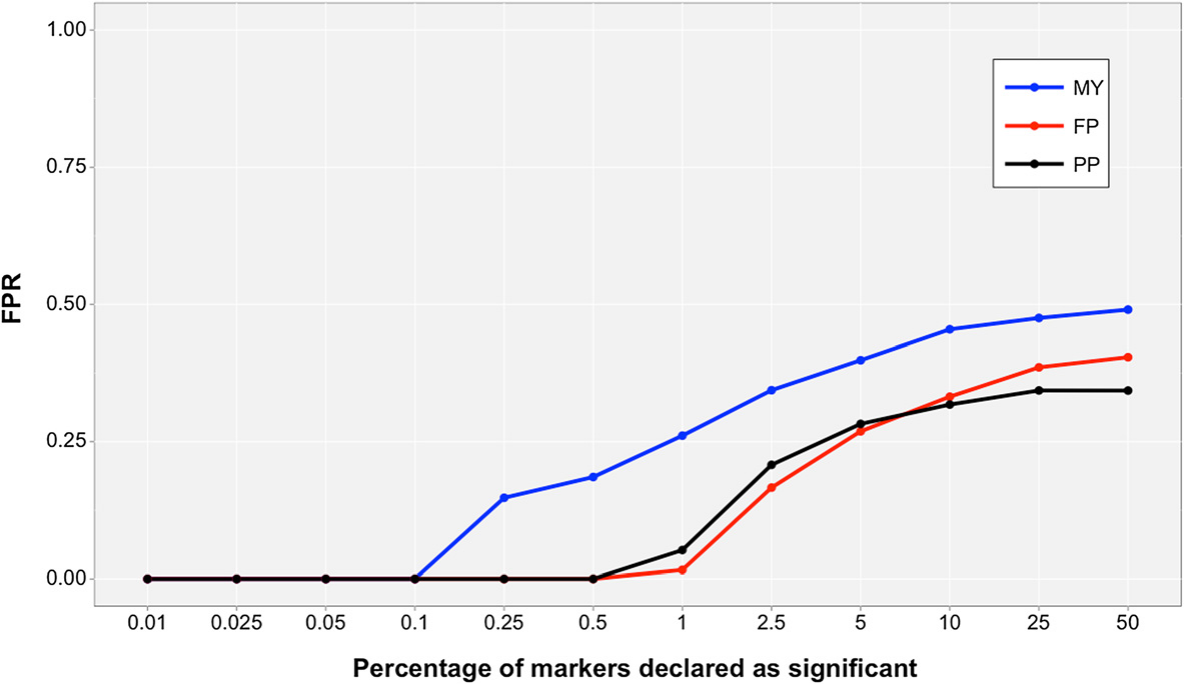
**Increase in false positive discoveries with increasing number of windows declared as significant in the GWAS.** The x-axis shows the number of windows declared significant in the association study, while the y-axis shows the corresponding False Positive Rate (FPR), i.e. the proportion of reported windows that did not contain an annotated QTL.

Figure 1 reports the sensitivity of the FPR to the increase in windows declared as significant for MY, FP and PP. In this study, regions that were found to have a strong impact on the genetic variance of the traits were consistent with those reported in the literature: for MY, the top 0.1% windows had at least one annotated QTL (all windows declared significant contained an annotated QTL) and only 25% of the top 1% windows contained false positives; for FP and PP, top 0.5% of windows had a QTL annotated, and slightly more than 25% of the top 5% windows did not have any annotated QTL. Thus, results from association analyses did not appear to be spurious. This indicates that markers that were most heavily weighted within the **wG** matrix were actually close to QTL for the traits of interest, while markers that were less heavily weighted still contributed to genomic relatedness, but with very low emphasis.

**Figure 2:**
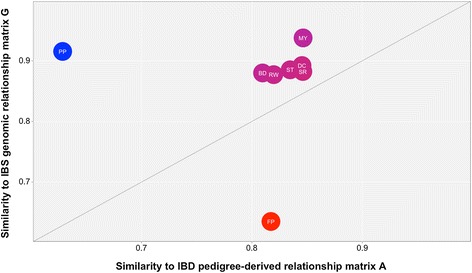
**Similarity between the weighted genomic matrix (wG) and the regular genomic (G) and pedigree-derived (A) relationship matrices.** Similarity is measured as the probability that two individuals that share the same medoid in a matrix and share the same medoid in the other. The relative position on the x-axis measures the similarity to **A** and the y-axis the similarity to **G**.

Mean, standard deviation and correlation with **G**^**BASE**^ for all genomic matrices computed are reported in Table S1 [See Additional file 1: Table S1]. The comparison of **wG** obtained with RR to that obtained with **A** and **G**^**BASE**^ is in Figure 2. All traits are represented in the plot by a circle (except for MC, which was omitted since it overlapped with MY). The relative position of **wG** on the x-axis measures its similarity to **A** (i.e. how many pairs of individuals that belong to the same medoid in **A** also belong to the same medoid in **wG**, over the total number of pairs), while the respective position on the y-axis indicates its similarity to **G**^**BASE**^. Similarity between **G**^**BASE**^ and **A** was equal to 0.82 (value not plotted). This means that 82% of the pairs of individuals that were in the same medoid in **A** were also in the same medoid in **G**^**BASE**^. We did not observe any trait for which **wG** appeared to be equivalent to **G**^**BASE**^ or very similar to **A**.

For BD and RW, similarities of **wG** to **A** and **G**^**BASE**^ were equal to 0.82 and 0.88, respectively. For these two traits, we can infer that the weighting procedure had a moderate impact, and that the weighing procedure was not affected by population stratification. MY, DC, MC, ST, and SR similarities of **wG** to **G**^**BASE**^ were around 0.90 and those to **A** were around 0.85. The weighting procedure resulted in relatedness among individuals that weakly resembled the pedigree population stratification for these traits. For FP, the presence of a QTL of strong impact decreased the similarity of **wG** to **G**^**BASE**^ (0.63), but did not affect its similarity to **A**, which remained at 0.82. For PP, **wG** resulted in a similarity to **A** that was very different (0.63) but it was still only moderately similar to **G**^**BASE**^ (0.92).

There was no evidence that the weighting procedure reintroduced population stratification in the weighted genomic matrices. For all traits, **wG** was different from **G**^**BASE**^, although this dissimilarity was stronger for FP, which is the trait that presented the strongest deviation from the Fisherian assumptions.

**Figure 3:**
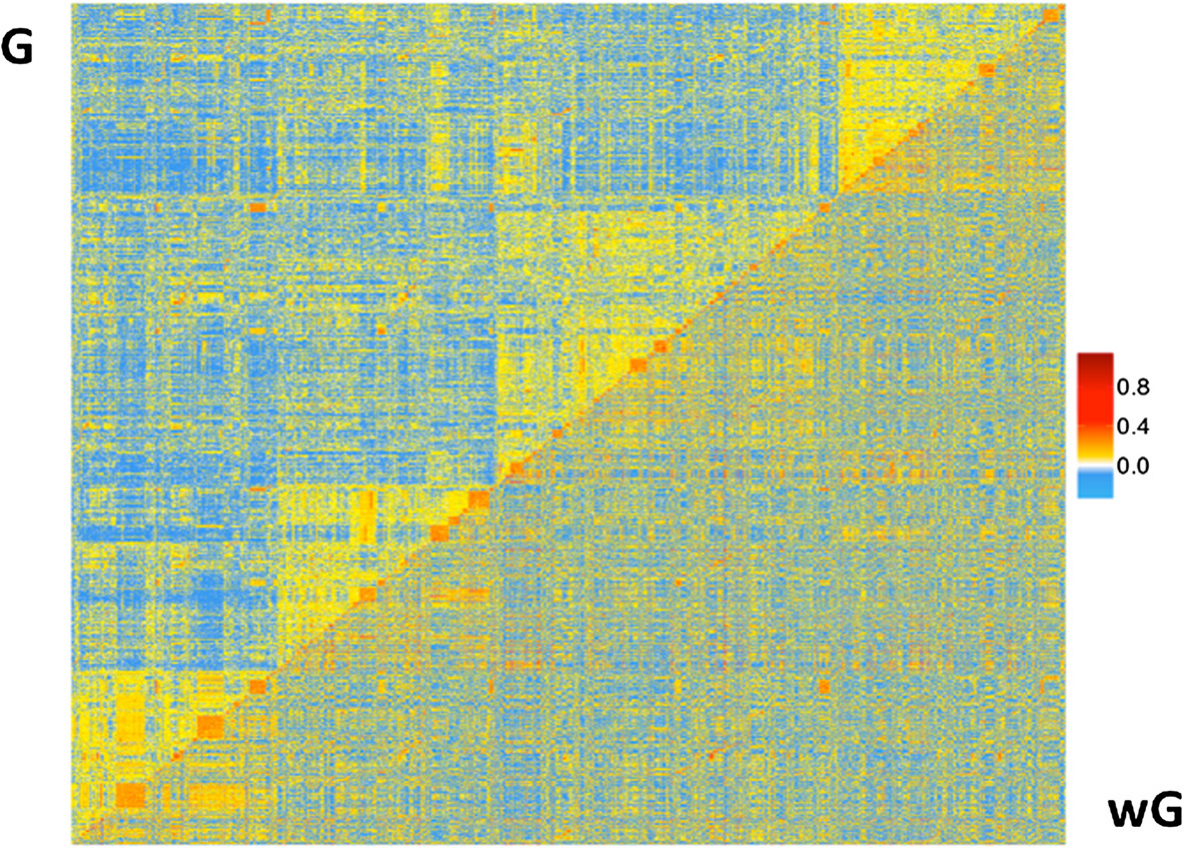
**Heatmap of the regular genomic matrix (upper triangle) and fat percentage weighted genomic matrix (lower triangle).** Warmer color indicates a positive relationship and colder color indicates a negative relationship.

In all cases, regardless of heritability and putative architecture of the traits, the weighted genomic matrices appeared to incorporate some signal other than population structure. We believe that this signal represents true QTL effects, which could be represented as a third dimension in Figure 2. The lack of knowledge about the true causative mutation(s) that determine the genetic variation of the trait did not allow us to draw this third dimension in our graphical representation. Nonetheless, the heatmap of **G**^**BASE**^ and **G**^**RR**^ for FP is displayed in Figure 3, with **G**^**RR**^ in the lower triangle, and **G**^**BASE**^ in the upper triangle (individuals were ordered by pedigree, i.e. were grouped if they shared the same medoid, as computed from the **A** matrix, as well as the same parents). Figure 3 shows a clear clustering of individuals occurring in **G**^**BASE**^, which clearly represents the four medoids. In **G**^**RR**^, the same clustering appears rearranged.

**Figure 4:**
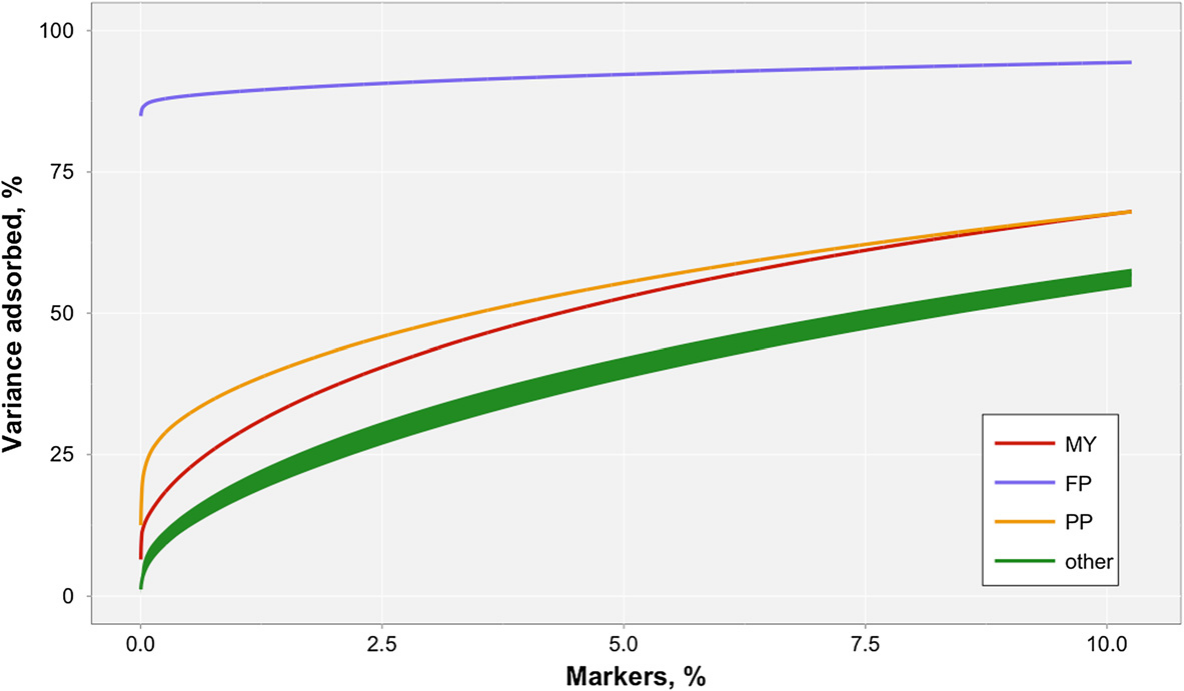
**Proportion of variance absorbed by top windows for milk yield (MY), fat percentage (FP), protein percentage (PP) and other traits represented as a single group (‘other’).**

The proportion of the variance absorbed by the top 10% of markers for each trait, as estimated with RR, is in Figure 4. As expected, FP presented a single region on chromosome 14 that absorbed a large part of the total marker variance. All other traits presented QTL of lesser magnitude. For MY and PP, the top 10% of markers absorbed approximately 60% of the genomic variance, and less than 0.1% of the markers absorbed about 25% of total genetic variance for PP and 15% for MY. The other traits (DC, MC, BD, RW, ST, SR) presented the same trend and were reported as a single line. For those traits, the top 0.1% of markers absorbed ~5% of genomic variance, and the top 10% about 50% of genomic variance. In summary, different genetic architectures can be seen across the traits. FP is affected by a QTL with large effect and PP and MY have few relatively large-effect QTL. The genetic architecture of other traits was relatively polygenic. Manhattan plots for the variance of 10-marker moving windows, as obtained with RR, are in Figures S1, S2, S3, S4, S5, S6, S7, S8, S9 and S10 [See Additional file 2]. Values are expressed as the proportion of genomic variance, computed as the sum of total marker variance regardless of the variance absorbed from pedigree-derived additive genetic variance. Windows with the greatest impact were located on chromosome 14 for FP and MY, as also reported by Grisart et al. [31], and large-impact windows were located on chromosome 18 for calving ease and type traits, in agreement with Cole et al. [32].

### Model predictive performance

**Figure 5:**
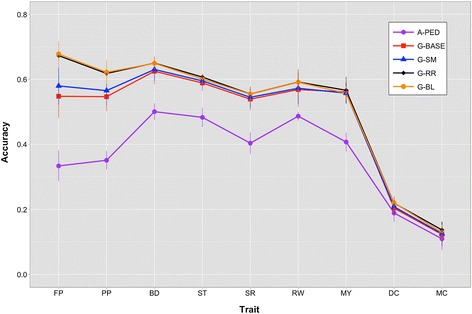
**Accuracy of prediction for traits analyzed with the different relationship matrices implemented in BLUP.** MY = milk yield, FP = fat percentage, PP = protein percentage, DC = direct calving ease, MC = maternal calving ease, BD = body depth, RW = rump width), ST = stature, and SR = strength; **A** ^**PED**^ = pedigree-derived, **G** ^**BASE**^ = base marker-derived genomic matrix, **G** ^**SM**^ = single-marker regression weighted genomic matrix, **G** ^**RR**^ = Ridge Regression weighted genomic matrix, **G** ^**BL**^ = Bayesian LASSO weighted genomic matrix.

**Figure 6:**
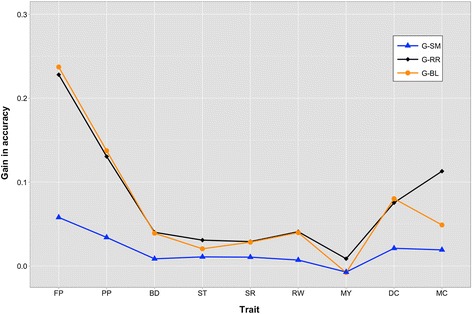
**Gain in accuracy of prediction over the regular genomic matrix for single-marker regression weighted genomic matrix (G** ^**SM**^ **), Ridge Regression weighted genomic matrix (G** ^**RR**^ **), Bayesian LASSO weighted genomic matrix (G** ^**BL**^ **).** Traits were analyzed with the different relationship matrices implemented in BLUP and included milk yield (MY), fat percentage (FP), protein percentage (PP), direct calving ease (DC), maternal calving ease (MC), body depth (BD), rump width (RW), stature (ST), and strength (SR).

**Figure 7:**
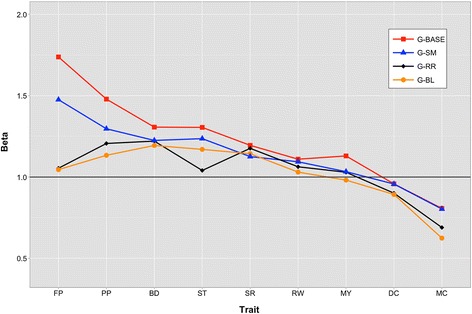
**Bias of prediction for traits analyzed with the different genomic relationship matrices implemented in BLUP.** MY = milk yield, FP = fat percentage, PP = protein percentage, DC = direct calving ease, MC = maternal calving ease, BD = body depth, RW = rump width), ST = stature, and SR = strength; **A** ^**PED**^ = pedigree-derived, **G** ^**BASE**^ = base marker-derived genomic matrix, **G** ^**SM**^ = single-marker regression weighted genomic matrix, **G** ^**RR**^ = Ridge Regression weighted genomic matrix, **G** ^**BL**^ = Bayesian LASSO weighted genomic matrix.

For ease of comparison of results, traits were clustered by heritability as high ‘H-h^2^’ (FP, PP, ST), medium ‘M-h^2^’ (BD, SR, RW, MY), and low ‘L-h^2^’ (DC, MC). This was done assuming that trait heritability was inversely proportional to the number of QTL. Although strong, this assumption facilitates the reporting and discussion of results, while verifying the assumption of better performance of **wG** models over **G**^**BASE**^ models. It is expected that improvement in performance is directly proportional to heritability and inversely proportional to the number of QTL. Accuracy of prediction of the different methods is in Figure 5. The marginal increase in accuracy with **wG** matrices relative to **G**^**BASE**^ is in Figure 6, while bias (the regression coefficient between predicted and observed) is presented in Figure 7. Tables containing the values reported in Figures S5, S6 and S7 are in Tables S2, S3 and S4 [See Additional file 3].

Prediction accuracy from **A**^**PED**^ was obtained for comparison purposes (Figure 5). It was high for MY, ST, RW, BD and SR (between 0.404 and 0.501), intermediate for PP and FP (0.351 and 0.334), and low for DC and MC (0.189 and 0.110). Models **G**^**BASE**^ and **G**^**SM**^ performed similarly across all traits, with a sizable advantage of **G**^**SM**^ only for FP. For FP, accuracies were 0.548 and 0.580 for **G**^**BASE**^ and **G**^**SM**^, respectively, while accuracies were 0.547 and 0.565 for PP. For other traits, no difference in accuracy was found between **G**^**BASE**^ and **G**^**SM**^.

Models **G**^**RR**^ and **G**^**BL**^ also performed similarly, and consistently better than other genomic models only for FP and PP. Accuracies were around 0.67 for FP, 0.62 for PP, 0.65 for BD, 0.60 for ST, 0.55 for SR, 0.59 for RW, 0.56 for MY, 0.22 for DC and 0.13 for MC. It is worth noting that poor predictive performance for DC and MC might be due to the relatively lower PTA reliabilities for these traits: de-regression of low-accuracy PTA may lead to over-inflation of the dePTA and therefore inflation of the residual variance in the association analysis. This reduced the relative gain achieved with genomic BLUP compared to pedigree-based BLUP for DC and MC. In addition, achieved prediction accuracy was weighted by the average PTA reliability in order to account for the lack of knowledge on true breeding values. This affected the results in terms of overall predictive ability for some traits: since PTA reliabilities were lower for DC and MC (see Table 1), the accuracy of prediction was reduced for all models.

The absolute gain in accuracy (Figure 6) that can be achieved from weighted genomic matrices appeared to depend highly on both heritability of the trait and the “putative” number of QTL, since **G**^**RR**^ and **G**^**BL**^ had higher accuracies than **G**^**BASE**^ only for H-h^2^. For FP and PP, there were few QTL that absorbed up to 25% of the total genomic variance. Other traits presented a large number of putative QTL with smaller effects. This resulted in relatively similar performances of **wG** and **G**^**BASE**^, as shown in Figure 2.

The relative gain in accuracy from **G**^**BASE**^ still appeared to depend on heritability. Relative gains in accuracy from **G**^**SM**^ ranged from −0.007 to 0.058 for MY and FP, respectively. In all cases, the gain obtained by **G**^**SM**^ was negligible, and feature selection based on *F*-test significance had little power to capture the genetic architecture of the traits. Moreover, gains from **G**^**RR**^ and **G**^**BL**^ ranged from 0.237 for FP to −0.008 for MY, both obtained with **G**^**BL**^. **G**^**RR**^ and **G**^**BL**^ achieved a relative gain in accuracy of 25% compared to **G**^**BASE**^ for FP, and of 15% for PP. For the other traits, the absolute increase with any of the weighted **G** matrices was small but prediction was consistently better than what could be obtained with **G**^**BASE**^.

While the absolute gain in predictive ability was proportional to heritability and the number of QTL, the relative gain that was achieved with weighting procedures was sizable in the case of low heritability, even with a QTL of moderate effect. This is of particular interest since traits with low heritability are limited in terms of the accuracy that could be obtained with any of the methods.

We found that bias of prediction was largely consistent with accuracy of prediction (Figure 7). **G**^**BASE**^ tended to inflate the variance of genetic merit of individuals, while reweighted **G**^**RR**^ and **G**^**BL**^ resulted in predictions that were the least biased, with **G**^**SM**^ showing intermediate performance. Differences between **G**^**BASE**^ and **wG** were stronger for H-h^2^: for FP, bias ranged from 1.738 for **G**^**BASE**^ to 1.046 for **G**^**BL**^; similarly, bias for PP ranged from 1.479 to 1.134 for **G**^**BASE**^ and **G**^**BL**^, respectively. Almost no difference in bias was reported for RW, since all methods seemed to give similar results (between 1.110 for **G**^**BASE**^ and 1.030 for **G**^**BL**^). Only **G**^**BASE**^ seemed to give relatively greater bias in predictions for MY (1.130 vs 0.981 for **G**^**BL**^) and SR (1.195 vs 1.126 for **G**^**SM**^) than **wG**. For BD, bias decreased from 1.305 for **G**^**BASE**^ to 1.040 for **G**^**RR**^, and for ST, bias also decreased from 1.308 for **G**^**BASE**^ to 1.194 for **G**^**BL**^. Surprisingly, the reversed situation was observed for MC, for which **G**^**RR**^ and **G**^**BL**^ resulted in a deflated variance of predictions (0.690 and 0.624, respectively), while **G**^**BASE**^ and **G**^**SM**^ were less biased predictors (0.807 and 0.803). Prediction appeared to be similar across models for DC, although with less bias (between 0.958 and 0.893 for **G**^**BASE**^ and **G**^**BL**^).

## Discussion

In this study, weighted GBLUP resulted in more accurate estimates of genomic values with lower bias in cross-validation for traits with high heritability and presence of QTL of even moderate effect. The largest decrease in bias from using weighted GBLUP was obtained for FP and PP, which presented the largest QTL, suggesting a greater departure of the genetic architecture from the Fisherian model.

The assumption that underlies **G**^**BASE**^ is that all markers contribute equally to genetic variation for the trait [8]. Based on real data, this has been verified to hold for traits under polygenic control. This study confirms that this assumption does not hold if the number of QTL is small (or smaller than the number of independent chromosome segments), especially with the presence of QTL with large effects [2]. For such traits, it is appropriate to give more weight to markers that are in stronger association with the QTL when constructing the genomic matrix, such that better predictions can be made. Our results are in agreement with those of Zhang et al. [12], who performed several simulations and found that weighted **G** matrices can yield gains in accuracy when the heritability of the trait is higher and the number of QTL is smaller. Subsequently, Wang et al. [14] demonstrated that weighted GBLUP can reduce bias and increase accuracy of prediction, outperforming regular GBLUP in simulated data. In another study, Zhang et al. [13] found that if **G** was informed with weights derived from several sources, Bayes-B seemed to outperform RR, however their results were still based on simulated data. With real data, de los Campos et al. [7] found that weighting marker contributions in the construction of **G** improved prediction for height in humans, which is a trait with high heritability and a large number of QTL [33]. Nonetheless, Legarra et al. [16] tested the predictive ability of LASSO-weighted GBLUP vs. regular GBLUP (as well as other methods) on French Holstein cattle and showed that weighted GBLUP can strongly improve predictive ability for traits such as FP, while the improvement was moderate for PP and null for MY, which is in complete agreement with the findings of our study. Investigating the predictive ability of GBLUP across Nordic Holstein and Nordic Red dairy cattle populations, Zhou et al. [34] compared different weighted genomic matrices to the regular one. However, no improvement was found when **G** was weighted with markers effects for any of the traits analyzed, which disagrees with our results.

Except for L-h^2^, any GBLUP outperformed **A**^**PED**^: the genomic relationship matrix resulted in higher accuracies than the pedigree-derived relationship matrix, in particular for traits with high heritability and a small number of QTL. This is in agreement with findings from Nejati-Javaremi et al. [3] and Hayes et al. [8], who compared genomic and pedigree-derived relationship matrices. The reason for the improved accuracy is that genomic information can capture variation in Mendelian sampling that occurs between individuals due to chromosomal recombination [5], because the number of independent chromosome segments is finite.

In this study, we tested different methods for deriving weights for **wG**. The least precise method for informing **G** appeared to include only markers with a P-value smaller than 0.05. The P-value was obtained from an F-test in a linear model in which the marker was considered as a fixed effect. Population stratification was corrected by comparing groups of paternal half-sibs (sire random additive genetic effect). In fact, this method, which was intentionally chosen as a ‘naive’ one, gave accuracies that were barely better than **G**^**BASE**^ and predictions were still biased. Thus, we can infer that this method would not be appropriate with our data. Other methods involved multiple regression on markers, with (BL) or without penalization (RR), that both considered the animal additive genetic effect to correct for stratification. These methods are known to be suitable for different genetic architectures since BL is sensitive to the genetic architecture of the trait, while RR is not. In fact, in this study, when GBLUP included information on marker realized variance from RR and BL, these methods were identical in terms of performance, both for accuracy and bias reduction. However, when marker effects were used to inform **G**, as computed with these methods, bias decreased and accuracy increased proportional to how much each trait differed from the assumption of complete polygenic architecture. We confirmed that predictions were not inflated by population stratification or founder effects. This was verified with two empirical approaches, and both lead to the conclusion that **wG** accounted for real QTL effects.

## Conclusions

Our study aimed at testing different sources of information in order to weight marker contributions into the genomic relationship matrix and relax the assumption of equal contributions from all markers to additive genetic variability among individuals. The methods used to estimate marker weights were single-marker regression (markers are included if the F-test is significant), Bayesian Ridge Regression and Bayesian LASSO. Results showed that the informed genomic matrices can yield higher accuracy and lower bias than the regular genomic matrix and the pedigree-derived relationship matrix. Prediction performance generally increased when the number of QTL for a given trait was small, which was the case for fat percentage and protein percentage. The weighted **G** matrices that yielded the overall best accuracies were those informed with realized marker effects from Ridge Regression and Bayesian LASSO, while discriminating markers based on their P-value led to average or null improvement of predictive performance. The increase in predictive performance compared to the traditional GBLUP for other traits was moderate, but for traits with low heritability and low pedigree-based prediction accuracy, informing the genomic matrix with realized marker variances appeared to be advantageous. Regular genomic matrices lean on the assumption that each marker contributes equally to the additive genetic relationship. We demonstrated that this assumption can be violated, with increases in predictive ability of GBLUP for traits for which contributions to genetic variability are not evenly distributed across genomic regions. It is worth noting that predictions were not inflated by population stratification or founder effects.

Our results could be useful to increase the prediction accuracy of selection candidates in dairy cattle breeding schemes that use genomic information and BLUP methodology. They could also serve as background in the search for different sources of information to weight marker contributions in the genomic matrix, and markers could be weighted relying on functional information about the annotated genes that lie in their proximity.

## Additional files

Additional file 1: Table S1. Mean, standard deviation and correlation with **G** ^**BASE**^ for all genomic matrices computed in this study. Description: G-VR II indicates the genomic matrix where markers are weighted by the reciprocal of their expected variance [11]. Additional file 2: Figure S1-S9. Manhattan plots of the proportion of genetic variance explained by 10-SNP moving windows for the traits analyzed (milk yield **(Figure S1)**, fat percentage **(Figure S2)**, protein percentage **(Figure S3)**, direct calving ease **(Figure S4)**, maternal calving ease **(Figure S5)**, body depth **(Figure S6)**, rump width **(Figure S7)**, stature **(Figure S8)**, strength **(Figure S9)**. Additional file 3: Tables S2-S4. Absolute accuracy, gain in accuracy and bias for the different models across the different traits analyzed.
