# Supplementary material for: Accounting for trait architecture in genomic predictions of US Holstein cattle using a weighted realized relationship matrix
Source: Genet Sel Evol. 2015 Apr 2;47(1):24. doi: 10.1186/s12711-015-0100-1 (PMC4381547; doi:10.1186/s12711-015-0100-1)
Supplement: Additional file 1: Table S1. — Mean, standard deviation and correlation with G BASE for all genomic matrices computed in this study. Description: G-VR II indicates the genomic matrix where markers are weighted by the reciprocal of their expected variance [11]. [file 12711_2015_100_MOESM1_ESM.pdf]

Table S1: Mean, standard deviation and correlation with $\mathbf{G}^{\mathbf{BASE}}$ for all genomic matrices computed in this study.

		Diagonal			Off-Diagonal		
		**Mean**	**SD**	**Corr**	**Mean**	**SD**	**Corr**
G-BASE		81.9*10e-2	3.02*10e-2	-	1.69*10-4	4.08*10e-2	-
G-VR II		99.1*10e-2	4.16*10e-2	0.961	-2.05*10e-4	4.81*10e-2	0.996
Milk Yield							
G-RR	2-3-4	85.108*10e-2	3.649*10e-2	0.848	1.756*10e-4	4.496*10e-2	0.920
G-RR	1-3-4	85.052*10e-2	3.55*10e-2	0.853	1.755*10e-4	4.461*10e-2	0.925
G-RR	1-2-4	85.184*10e-2	3.586*10e-2	0.863	1.758*10e-4	4.452*10e-2	0.928
G-RR	1-2-3	85.263*10e-2	3.496*10e-2	0.874	1.759*10e-4	4.441*10e-2	0.936
G-BL	2-3-4	83.197*10e-2	7.976*10e-2	0.423	1.717*10e-4	6.435*10e-2	0.614
G-BL	1-3-4	83.15*10e-2	8.766*10e-2	0.384	1.716*10e-4	7.028*10e-2	0.564
G-BL	1-2-4	83.272*10e-2	8.104*10e-2	0.410	1.718*10e-4	6.735*10e-2	0.594
G-BL	1-2-3	84.646*10e-2	5.942*10e-2	0.548	1.747*10e-4	5.729*10e-2	0.714
G-SMR	2-3-4	75.602*10e-2	3.015*10e-2	0.972	1.56*10e-4	3.773*10e-2	0.991
G-SMR	1-3-4	75.599*10e-2	3.007*10e-2	0.971	1.56*10e-4	3.787*10e-2	0.991
G-SMR	1-2-4	75.608*10e-2	3.051*10e-2	0.969	1.56*10e-4	3.785*10e-2	0.990
G-SMR	1-2-3	75.647*10e-2	3.012*10e-2	0.970	1.561*10e-4	3.772*10e-2	0.990
Fat Percentage							
G-RR	2-3-4	85.034*10e-2	8.948*10e-2	0.377	1.755*10e-4	8.43*10e-2	0.478
G-RR	1-3-4	85.231*10e-2	8.578*10e-2	0.390	1.759*10e-4	8.136*10e-2	0.497
G-RR	1-2-4	85.251*10e-2	9.004*10e-2	0.374	1.759*10e-4	8.536*10e-2	0.473
G-RR	1-2-3	85.209*10e-2	8.29*10e-2	0.406	1.758*10e-4	7.935*10e-2	0.512
G-BL	2-3-4	65.255*10e-2	75.352*10e-2	0.098	1.347*10e-4	48.945*10e-2	0.049
G-BL	1-3-4	65.191*10e-2	75.555*10e-2	0.098	1.345*10e-4	49.013*10e-2	0.049
G-BL	1-2-4	63.96*10e-2	79.779*10e-2	0.096	1.32*10e-4	51.742*10e-2	0.045
G-BL	1-2-3	65.552*10e-2	74.331*10e-2	0.099	1.353*10e-4	48.287*10e-2	0.051
G-SMR	2-3-4	75.871*10e-2	3.143*10e-2	0.935	1.566*10e-4	3.894*10e-2	0.973
G-SMR	1-3-4	76.036*10e-2	3.099*10e-2	0.943	1.569*10e-4	3.88*10e-2	0.976
G-SMR	1-2-4	75.824*10e-2	3.145*10e-2	0.938	1.565*10e-4	3.873*10e-2	0.972
G-SMR	1-2-3	76.09*10e-2	3.107*10e-2	0.936	1.57*10e-4	3.881*10e-2	0.971
Protein Percentage							
G-RR	2-3-4	84.821*10e-2	3.778*10e-2	0.802	1.75*10e-4	4.623*10e-2	0.894
G-RR	1-3-4	84.882*10e-2	3.767*10e-2	0.803	1.752*10e-4	4.639*10e-2	0.894
G-RR	1-2-4	85.064*10e-2	3.821*10e-2	0.790	1.755*10e-4	4.658*10e-2	0.889
G-RR	1-2-3	85.002*10e-2	3.737*10e-2	0.799	1.754*10e-4	4.646*10e-2	0.895
G-BL	2-3-4	81.134*10e-2	13.219*10e-2	0.279	1.674*10e-4	9.535*10e-2	0.406
G-BL	1-3-4	81.028*10e-2	14.184*10e-2	0.265	1.672*10e-4	10.028*10e-2	0.385
G-BL	1-2-4	80.844*10e-2	15.668*10e-2	0.245	1.668*10e-4	10.857*10e-2	0.350
G-BL	1-2-3	83.295*10e-2	7.995*10e-2	0.413	1.719*10e-4	6.997*10e-2	0.580
G-SMR	2-3-4	76.613*10e-2	3.257*10e-2	0.887	1.581*10e-4	4.088*10e-2	0.946
G-SMR	1-3-4	76.614*10e-2	3.225*10e-2	0.895	1.581*10e-4	4.055*10e-2	0.953
G-SMR	1-2-4	76.742*10e-2	3.284*10e-2	0.870	1.584*10e-4	4.095*10e-2	0.943
G-SMR	1-2-3	76.543*10e-2	3.224*10e-2	0.885	1.58*10e-4	4.045*10e-2	0.948

Direct Calving Ease							
G-RR	2-3-4	85.234*10e-2	3.406*10e-2	0.889	1.759*10e-4	4.391*10e-2	0.946
G-RR	1-3-4	85.158*10e-2	3.441*10e-2	0.872	1.757*10e-4	4.424*10e-2	0.938
G-RR	1-2-4	85.323*10e-2	3.349*10e-2	0.889	1.761*10e-4	4.382*10e-2	0.948
G-RR	1-2-3	85.185*10e-2	3.42*10e-2	0.883	1.758*10e-4	4.404*10e-2	0.944
G-BL	2-3-4	84.866*10e-2	3.977*10e-2	0.774	1.751*10e-4	4.596*10e-2	0.895
G-BL	1-3-4	84.175*10e-2	4.715*10e-2	0.659	1.737*10e-4	4.843*10e-2	0.838
G-BL	1-2-4	85.22*10e-2	3.61*10e-2	0.829	1.759*10e-4	4.493*10e-2	0.921
G-BL	1-2-3	84.587*10e-2	4.08*10e-2	0.753	1.745*10e-4	4.612*10e-2	0.889
G-SMR	2-3-4	75.424*10e-2	2.91*10e-2	0.986	1.556*10e-4	3.738*10e-2	0.997
G-SMR	1-3-4	75.478*10e-2	2.91*10e-2	0.987	1.558*10e-4	3.752*10e-2	0.997
G-SMR	1-2-4	75.457*10e-2	2.911*10e-2	0.986	1.557*10e-4	3.747*10e-2	0.996
G-SMR	1-2-3	75.455*10e-2	2.904*10e-2	0.986	1.557*10e-4	3.752*10e-2	0.997
Maternal Calving Ease							
G-RR	2-3-4	85.362*10e-2	3.281*10e-2	0.913	1.761*10e-4	4.327*10e-2	0.958
G-RR	1-3-4	85.376*10e-2	3.28*10e-2	0.906	1.762*10e-4	4.338*10e-2	0.955
G-RR	1-2-4	85.38*10e-2	3.277*10e-2	0.915	1.762*10e-4	4.336*10e-2	0.959
G-RR	1-2-3	85.315*10e-2	3.315*10e-2	0.909	1.761*10e-4	4.357*10e-2	0.957
G-BL	2-3-4	85.345*10e-2	3.346*10e-2	0.893	1.761*10e-4	4.364*10e-2	0.948
G-BL	1-3-4	85.305*10e-2	3.35*10e-2	0.885	1.76*10e-4	4.365*10e-2	0.946
G-BL	1-2-4	85.597*10e-2	4.925*10e-2	0.610	1.766*10e-4	5.638*10e-2	0.742
G-BL	1-2-3	85.414*10e-2	4.063*10e-2	0.734	1.763*10e-4	4.899*10e-2	0.851
G-SMR	2-3-4	75.436*10e-2	2.903*10e-2	0.987	1.557*10e-4	3.745*10e-2	0.997
G-SMR	1-3-4	75.507*10e-2	2.89*10e-2	0.988	1.558*10e-4	3.75*10e-2	0.997
G-SMR	1-2-4	75.368*10e-2	2.901*10e-2	0.987	1.555*10e-4	3.744*10e-2	0.997
G-SMR	1-2-3	75.474*10e-2	2.896*10e-2	0.988	1.557*10e-4	3.755*10e-2	0.997
Stature							
G-RR	2-3-4	85.111*10e-2	3.343*10e-2	0.910	1.756*10e-4	4.341*10e-2	0.955
G-RR	1-3-4	85.297*10e-2	3.395*10e-2	0.901	1.76*10e-4	4.383*10e-2	0.950
G-RR	1-2-4	85.292*10e-2	3.315*10e-2	0.908	1.76*10e-4	4.356*10e-2	0.955
G-RR	1-2-3	85.121*10e-2	3.355*10e-2	0.901	1.757*10e-4	4.352*10e-2	0.951
G-BL	2-3-4	85.108*10e-2	3.739*10e-2	0.806	1.756*10e-4	4.585*10e-2	0.900
G-BL	1-3-4	85.326*10e-2	3.711*10e-2	0.819	1.761*10e-4	4.596*10e-2	0.905
G-BL	1-2-4	85.35*10e-2	3.54*10e-2	0.849	1.761*10e-4	4.491*10e-2	0.924
G-BL	1-2-3	85.118*10e-2	4.598*10e-2	0.648	1.756*10e-4	5.149*10e-2	0.797
G-SMR	2-3-4	75.869*10e-2	3.042*10e-2	0.964	1.566*10e-4	3.851*10e-2	0.985
G-SMR	1-3-4	75.786*10e-2	3.054*10e-2	0.963	1.564*10e-4	3.85*10e-2	0.985
G-SMR	1-2-4	75.78*10e-2	3.081*10e-2	0.959	1.564*10e-4	3.865*10e-2	0.983
G-SMR	1-2-3	75.734*10e-2	3.066*10e-2	0.959	1.563*10e-4	3.849*10e-2	0.983
Strenght							
G-RR	2-3-4	85.161*10e-2	3.374*10e-2	0.903	1.757*10e-4	4.353*10e-2	0.951
G-RR	1-3-4	85.385*10e-2	3.347*10e-2	0.898	1.762*10e-4	4.381*10e-2	0.949
G-RR	1-2-4	85.207*10e-2	3.342*10e-2	0.906	1.758*10e-4	4.346*10e-2	0.952
G-RR	1-2-3	85.146*10e-2	3.39*10e-2	0.895	1.757*10e-4	4.379*10e-2	0.947
G-BL	2-3-4	85.097*10e-2	4.167*10e-2	0.731	1.756*10e-4	4.82*10e-2	0.852
G-BL	1-3-4	85.34*10e-2	3.856*10e-2	0.783	1.761*10e-4	4.651*10e-2	0.889
G-BL	1-2-4	85.208*10e-2	3.818*10e-2	0.794	1.758*10e-4	4.617*10e-2	0.892

G-BL	1-2-3	85.079*10e-2	4.825*10e-2	0.627	1.756*10e-4	5.274*10e-2	0.776
G-SMR	2-3-4	75.767*10e-2	2.97*10e-2	0.972	1.563*10e-4	3.802*10e-2	0.990
G-SMR	1-3-4	75.683*10e-2	2.994*10e-2	0.969	1.562*10e-4	3.801*10e-2	0.990
G-SMR	1-2-4	75.564*10e-2	3.001*10e-2	0.967	1.559*10e-4	3.788*10e-2	0.988
G-SMR	1-2-3	75.763*10e-2	2.98*10e-2	0.970	1.563*10e-4	3.801*10e-2	0.990
Body Depth							
G-RR	2-3-4	85.098*10e-2	3.414*10e-2	0.892	1.756*10e-4	4.369*10e-2	0.944
G-RR	1-3-4	85.285*10e-2	3.421*10e-2	0.890	1.76*10e-4	4.4*10e-2	0.944
G-RR	1-2-4	85.263*10e-2	3.388*10e-2	0.897	1.759*10e-4	4.386*10e-2	0.948
G-RR	1-2-3	85.095*10e-2	3.447*10e-2	0.886	1.756*10e-4	4.404*10e-2	0.941
G-BL	2-3-4	84.84*10e-2	4.596*10e-2	0.672	1.751*10e-4	5.005*10e-2	0.814
G-BL	1-3-4	84.843*10e-2	4.315*10e-2	0.721	1.751*10e-4	4.795*10e-2	0.855
G-BL	1-2-4	85.166*10e-2	3.902*10e-2	0.785	1.757*10e-4	4.647*10e-2	0.888
G-BL	1-2-3	84.778*10e-2	5.236*10e-2	0.588	1.749*10e-4	5.464*10e-2	0.745
G-SMR	2-3-4	75.974*10e-2	2.976*10e-2	0.968	1.568*10e-4	3.837*10e-2	0.987
G-SMR	1-3-4	75.867*10e-2	3.006*10e-2	0.968	1.566*10e-4	3.827*10e-2	0.988
G-SMR	1-2-4	75.626*10e-2	3.004*10e-2	0.964	1.561*10e-4	3.825*10e-2	0.986
G-SMR	1-2-3	75.859*10e-2	2.992*10e-2	0.967	1.565*10e-4	3.831*10e-2	0.987
Rump Width							
G-RR	2-3-4	85.024*10e-2	3.357*10e-2	0.896	1.755*10e-4	4.34*10e-2	0.948
G-RR	1-3-4	85.078*10e-2	3.362*10e-2	0.898	1.756*10e-4	4.353*10e-2	0.950
G-RR	1-2-4	85.211*10e-2	3.337*10e-2	0.906	1.758*10e-4	4.351*10e-2	0.952
G-RR	1-2-3	85.052*10e-2	3.372*10e-2	0.891	1.755*10e-4	4.353*10e-2	0.946
G-BL	2-3-4	84.98*10e-2	3.799*10e-2	0.792	1.754*10e-4	4.601*10e-2	0.892
G-BL	1-3-4	85*10e-2	3.715*10e-2	0.810	1.754*10e-4	4.55*10e-2	0.906
G-BL	1-2-4	85.158*10e-2	3.773*10e-2	0.808	1.757*10e-4	4.59*10e-2	0.902
G-BL	1-2-3	84.867*10e-2	4.086*10e-2	0.731	1.751*10e-4	4.763*10e-2	0.860
G-SMR	2-3-4	75.493*10e-2	2.925*10e-2	0.977	1.558*10e-4	3.77*10e-2	0.993
G-SMR	1-3-4	75.484*10e-2	2.924*10e-2	0.976	1.558*10e-4	3.769*10e-2	0.992
G-SMR	1-2-4	75.368*10e-2	2.949*10e-2	0.973	1.555*10e-4	3.76*10e-2	0.992
G-SMR	1-2-3	75.555*10e-2	2.928*10e-2	0.976	1.559*10e-4	3.773*10e-2	0.992
