# Supplementary material for: Accounting for trait architecture in genomic predictions of US Holstein cattle using a weighted realized relationship matrix
Source: Genet Sel Evol. 2015 Apr 2;47(1):24. doi: 10.1186/s12711-015-0100-1 (PMC4381547; doi:10.1186/s12711-015-0100-1)
Supplement: Additional file 2: Figure S1-S9. — Manhattan plots of the proportion of genetic variance explained by 10-SNP moving windows for the traits analyzed (milk yield (Figure S1), fat percentage (Figure S2), protein percentage (Figure S3), direct calving ease (Figure S4), maternal calving ease (Figure S5), body depth (Figure S6), rump width (Figure S7), stature (Figure S8), strength (Figure S9). [file 12711_2015_100_MOESM2_ESM.pdf]

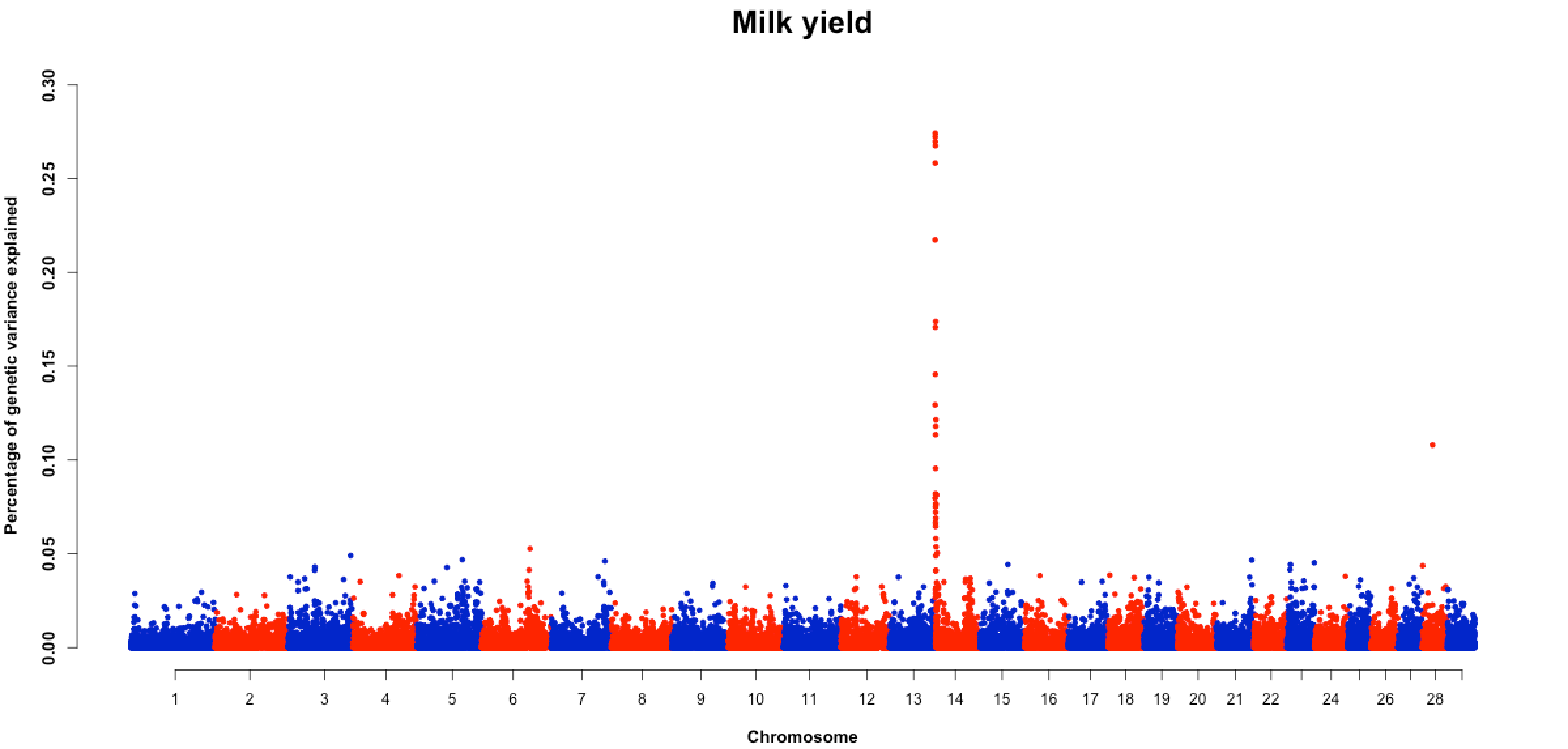

Milk yield
Percentage of genetic variance explained
0.00
0.05
0.10
0.15
0.20
0.25
0.30
1
2
3
4
5
6
7
8
9
10
11
12
13
14
15
16
17
18
19
20
21
22
24
26
28
Chromosome

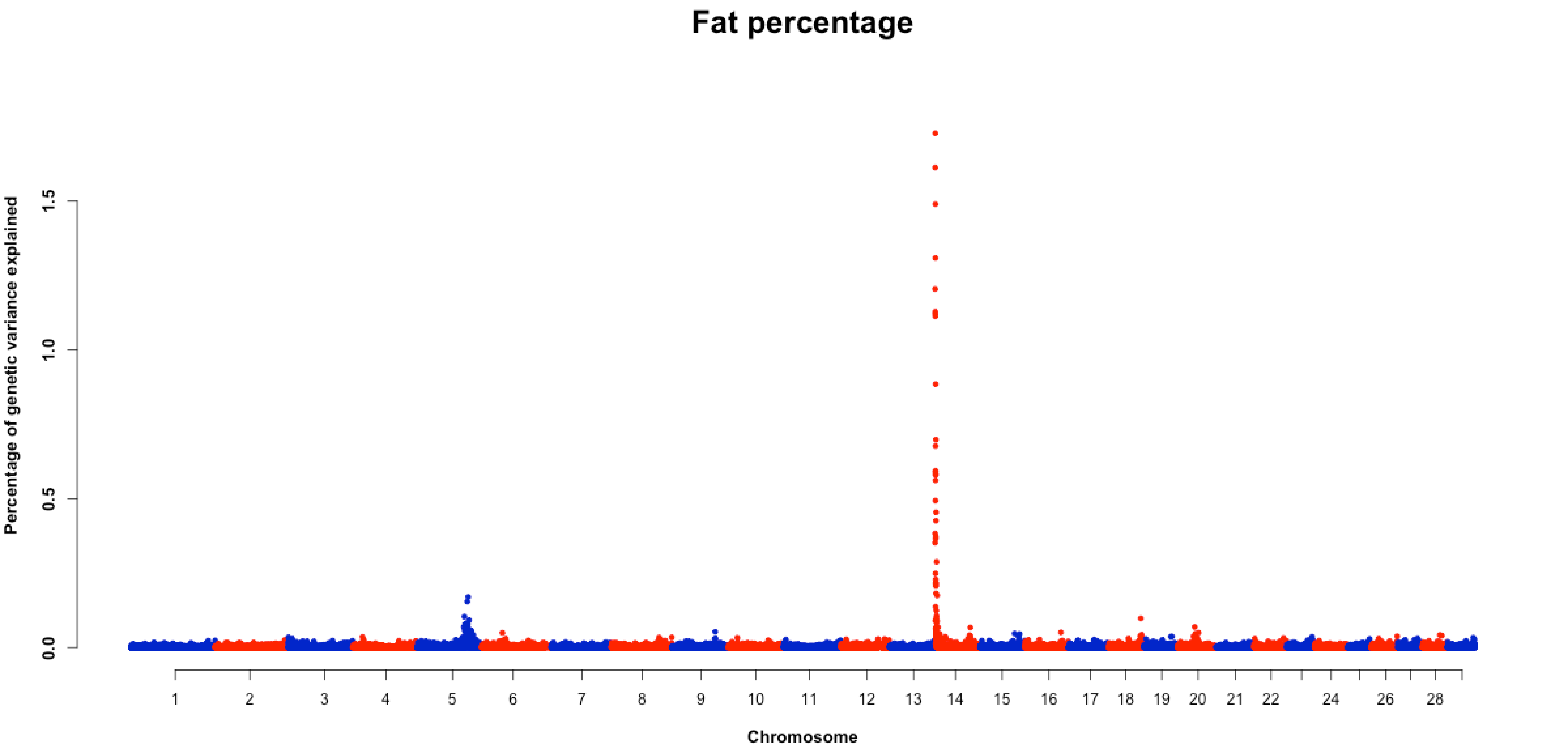

Fat percentage
Percentage of genetic variance explained
0.0
0.5
1.0
1.5
1
2
3
4
5
6
7
8
9
10
11
12
13
14
15
16
17
18
19
20
21
22
24
26
28
Chromosome

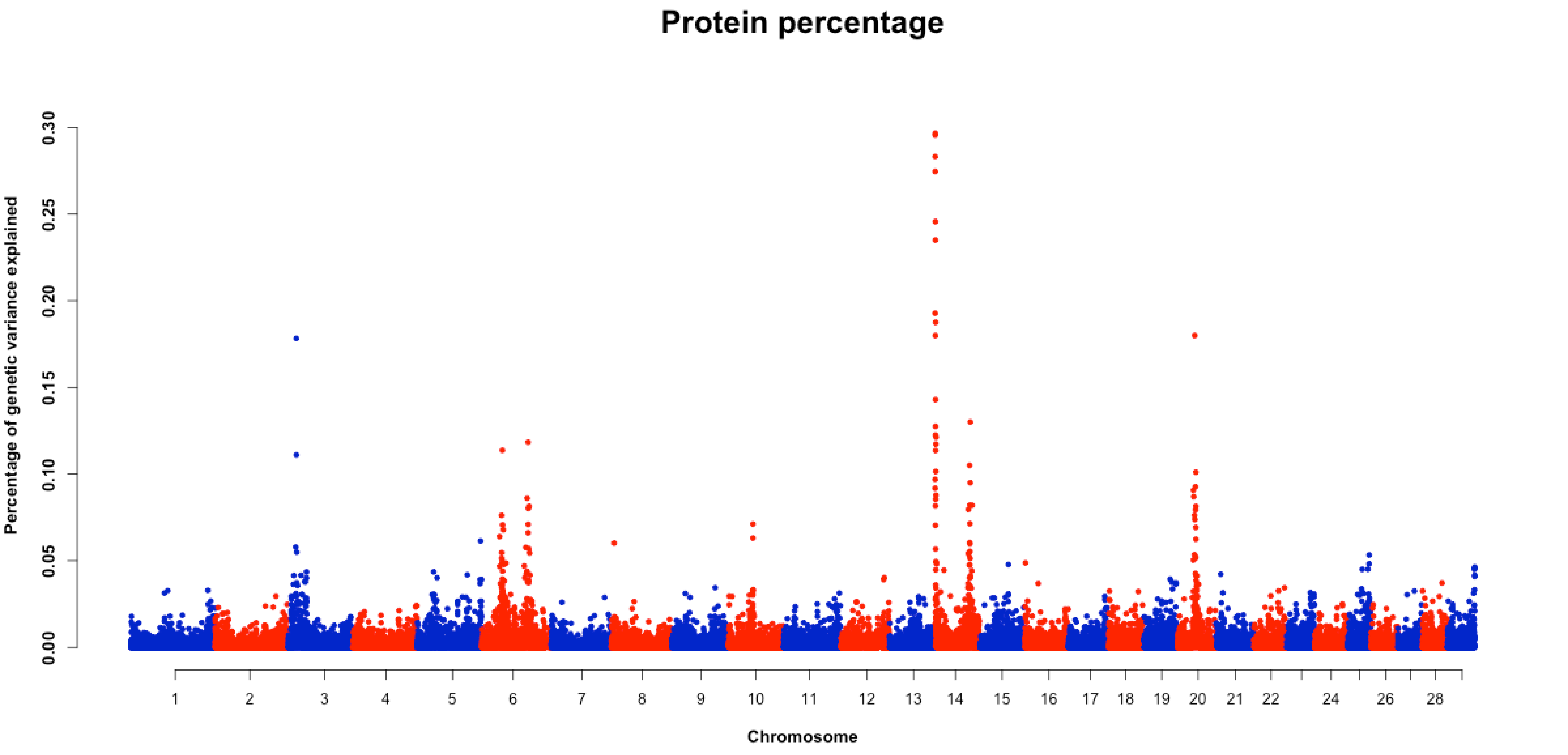

Protein percentage
Percentage of genetic variance explained
0.00
0.05
0.10
0.15
0.20
0.25
0.30
Chromosome
1
2
3
4
5
6
7
8
9
10
11
12
13
14
15
16
17
18
19
20
21
22
24
26
28

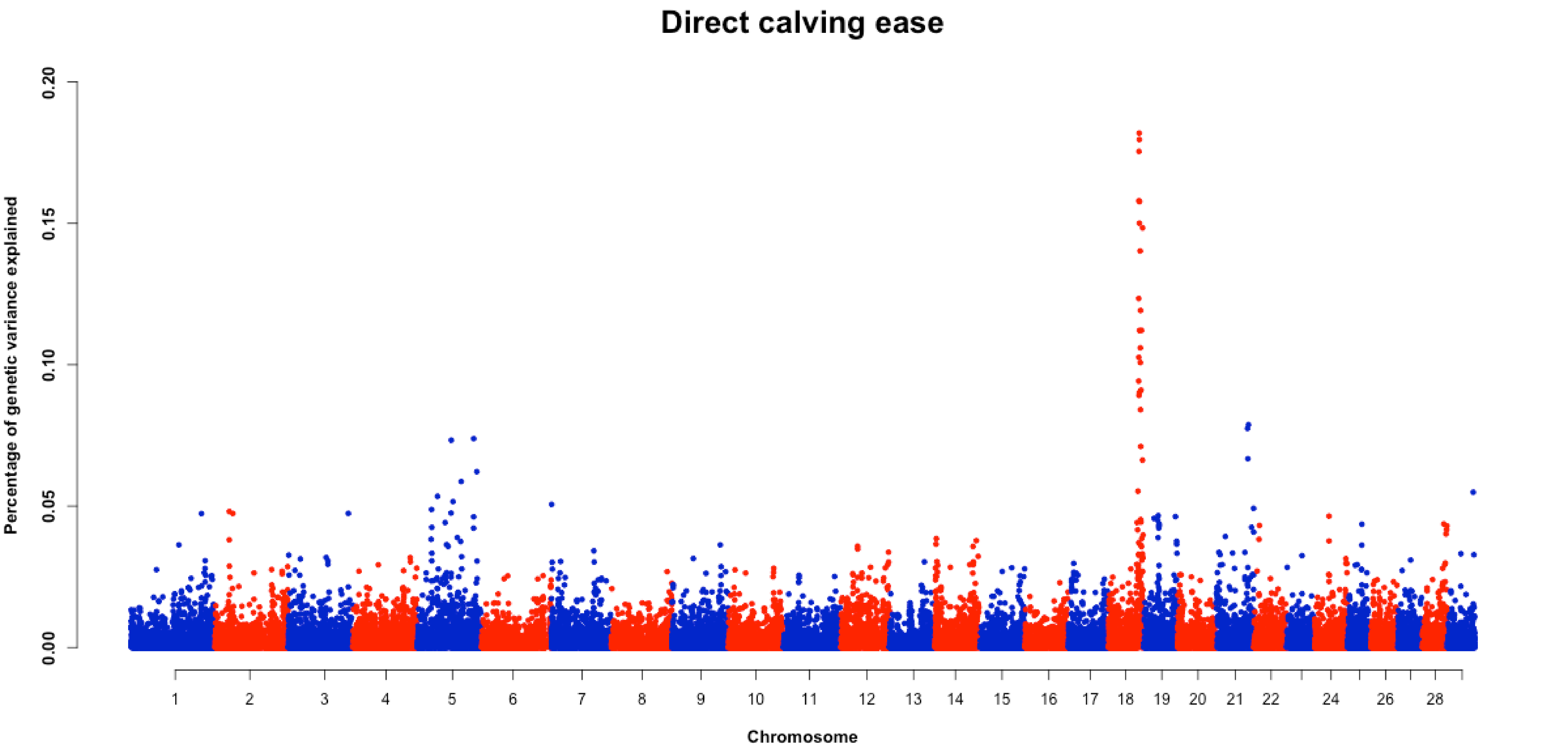
Direct calving ease
Percentage of genetic variance explained
0.00
0.05
0.10
0.15
0.20
1
2
3
4
5
6
7
8
9
10
11
12
13
14
15
16
17
18
19
20
21
22
24
26
28
Chromosome

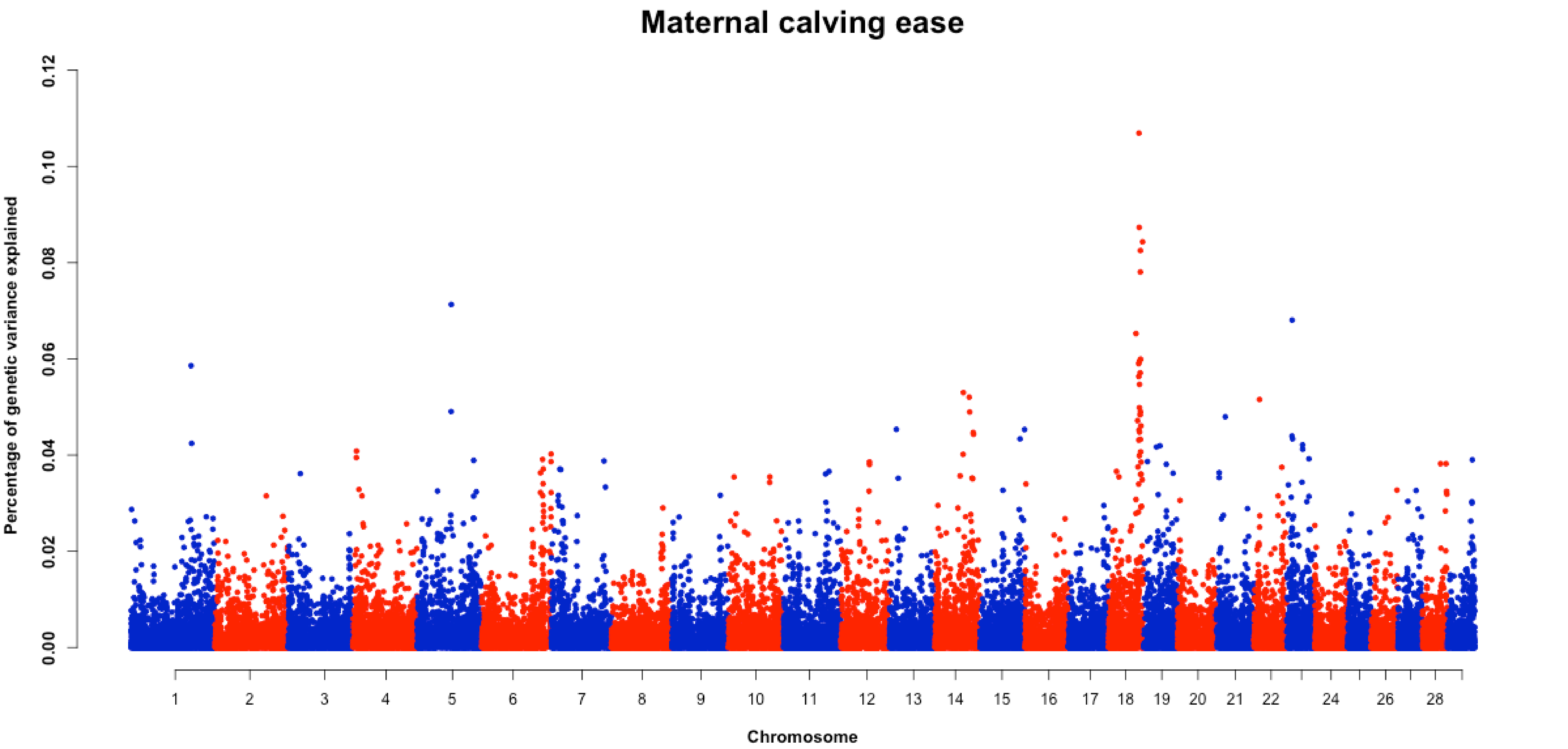
Maternal calving ease
Percentage of genetic variance explained
0.00
0.02
0.04
0.06
0.08
0.10
0.12
1
2
3
4
5
6
7
8
9
10
11
12
13
14
15
16
17
18
19
20
21
22
24
26
28
Chromosome

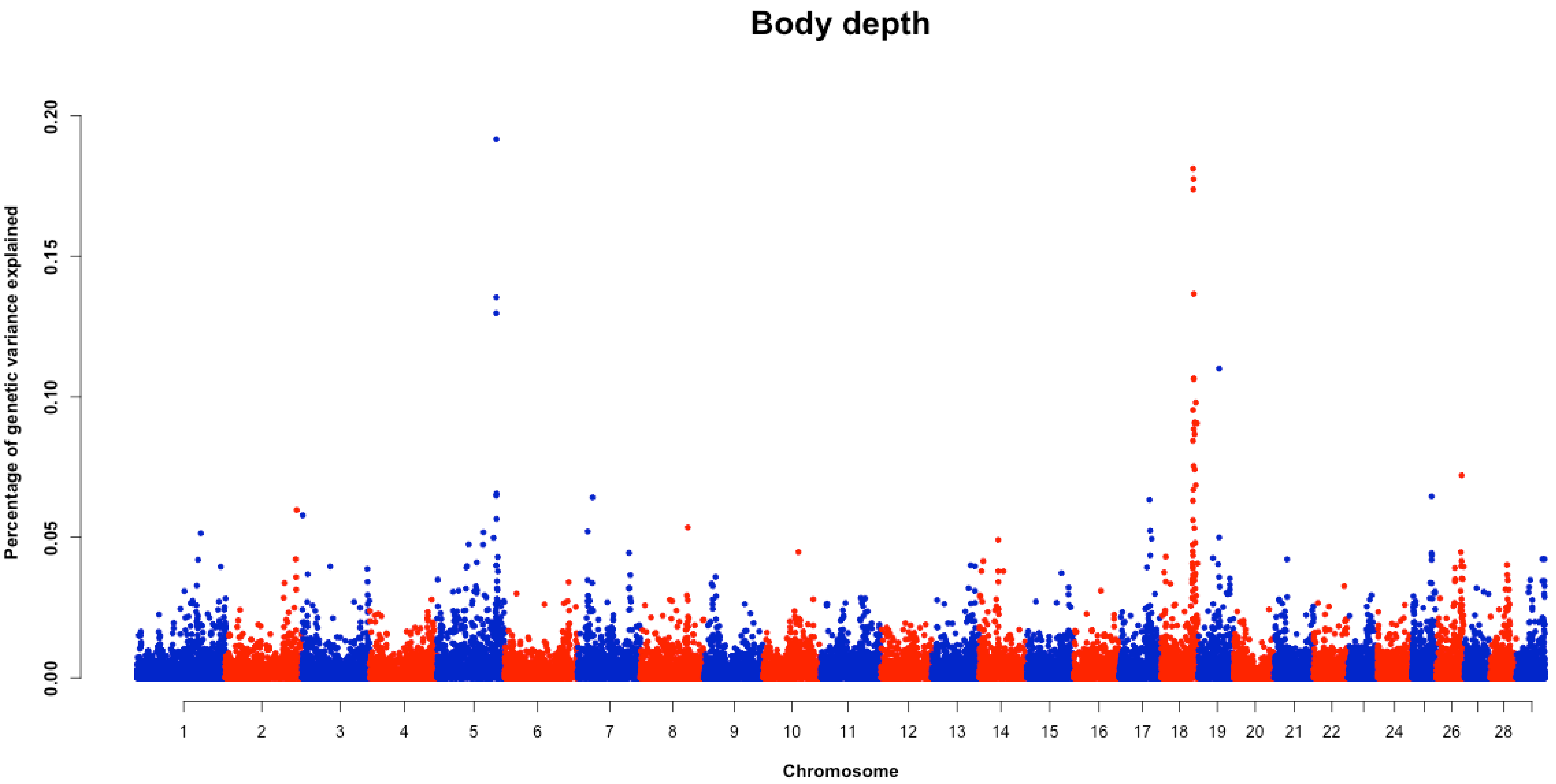

Body depth
Percentage of genetic variance explained
0.00
0.05
0.10
0.15
0.20
Chromosome
1
2
3
4
5
6
7
8
9
10
11
12
13
14
15
16
17
18
19
20
21
22
24
26
28

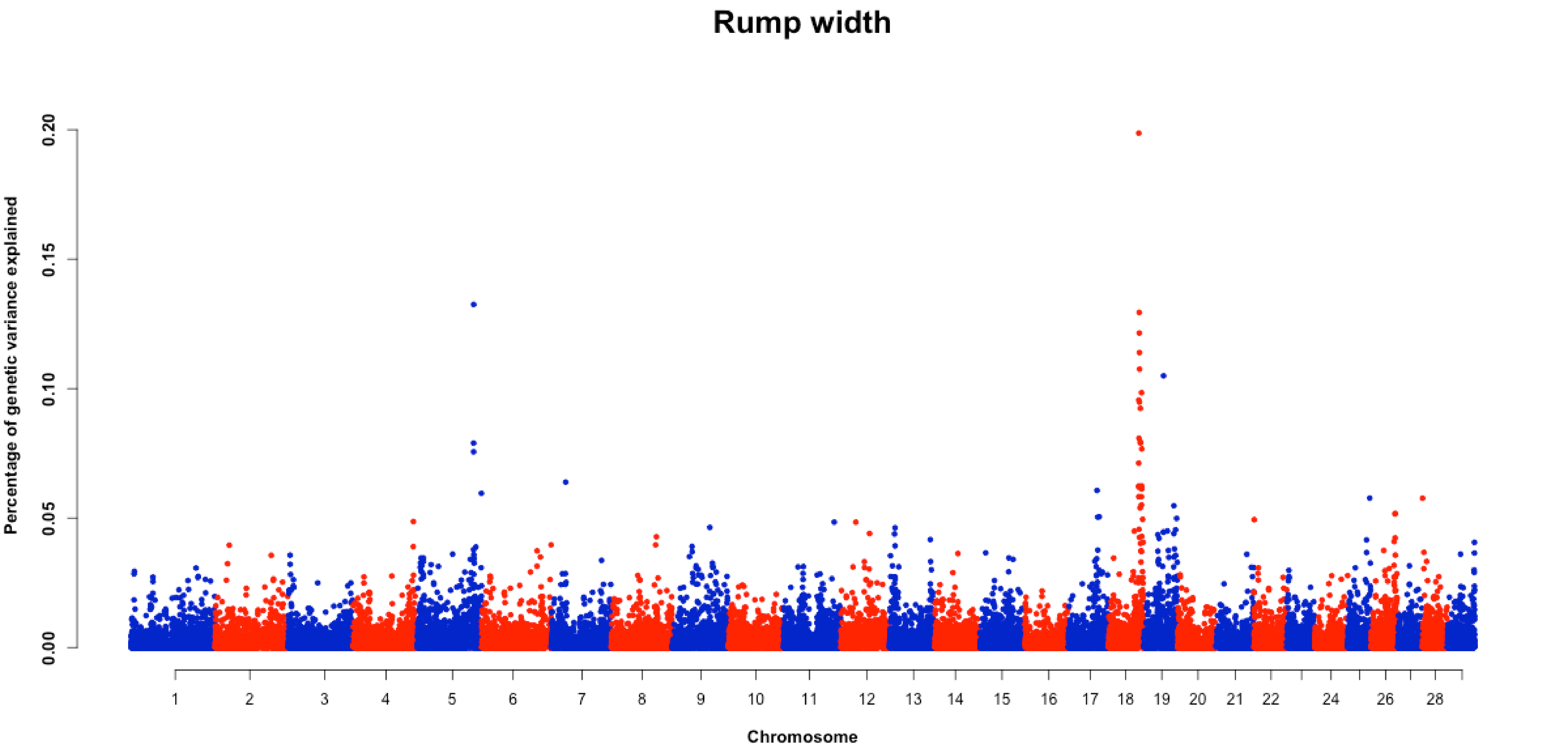
Rump width
Percentage of genetic variance explained
0.00
0.05
0.10
0.15
0.20
Chromosome
1
2
3
4
5
6
7
8
9
10
11
12
13
14
15
16
17
18
19
20
21
22
24
26
28

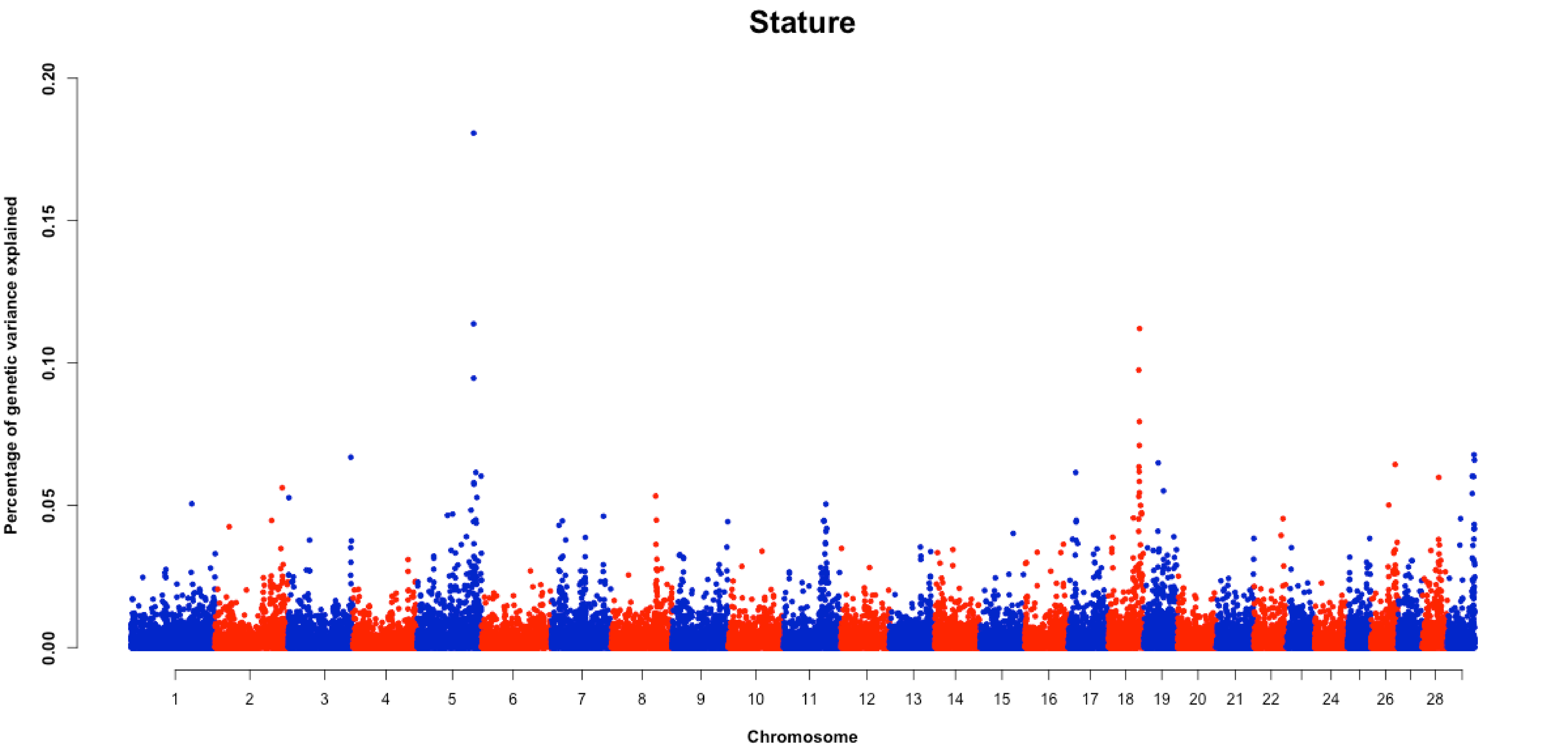

Stature
Percentage of genetic variance explained
0.00
0.05
0.10
0.15
0.20
Chromosome
1
2
3
4
5
6
7
8
9
10
11
12
13
14
15
16
17
18
19
20
21
22
24
26
28

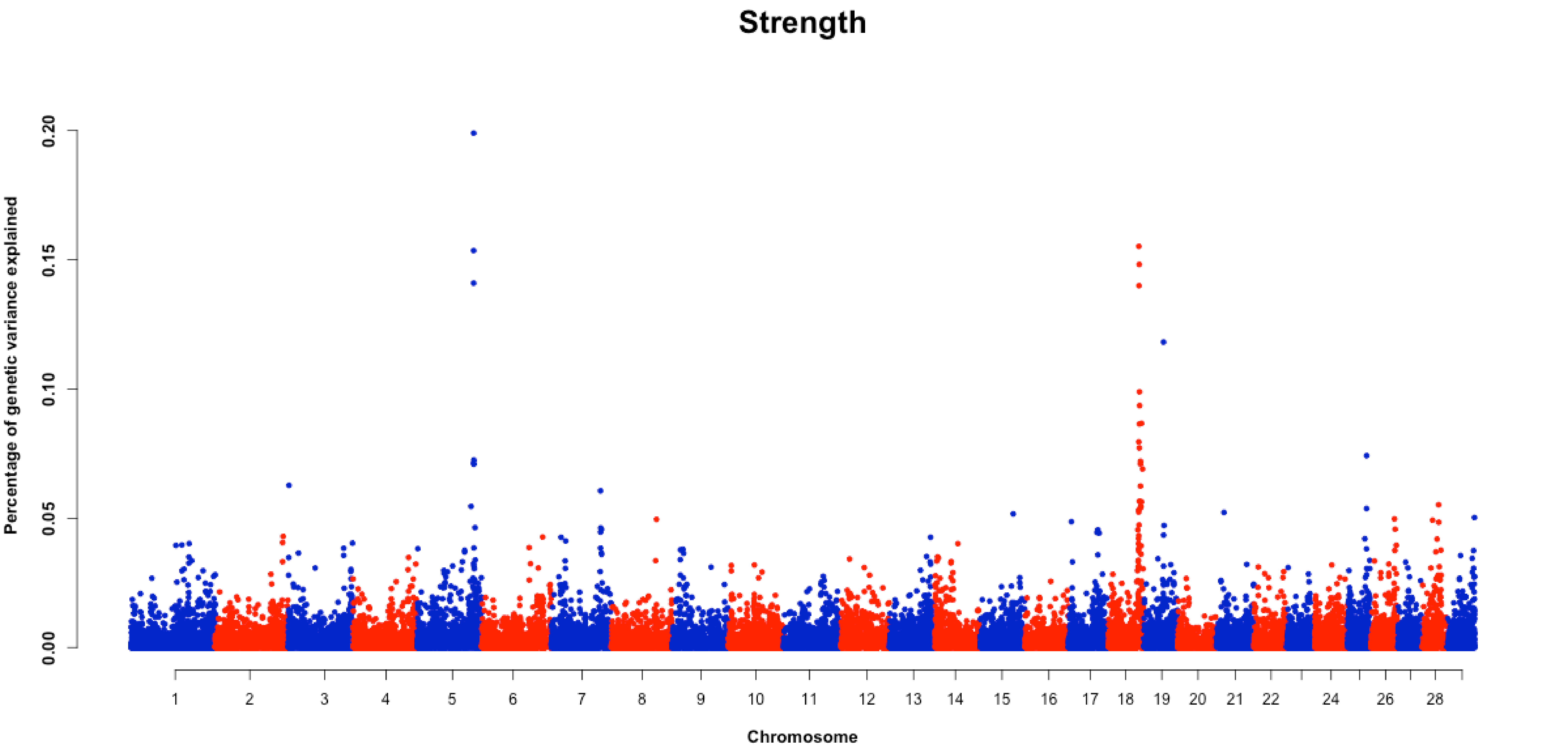

Strength
Percentage of genetic variance explained
0.00
0.05
0.10
0.15
0.20
1
2
3
4
5
6
7
8
9
10
11
12
13
14
15
16
17
18
19
20
21
22
24
26
28
Chromosome
