# Supplementary material for: Accounting for trait architecture in genomic predictions of US Holstein cattle using a weighted realized relationship matrix
Source: Genet Sel Evol. 2015 Apr 2;47(1):24. doi: 10.1186/s12711-015-0100-1 (PMC4381547; doi:10.1186/s12711-015-0100-1)
Supplement: Additional file 3: Tables S2-S4. — Absolute accuracy, gain in accuracy and bias for the different models across the different traits analyzed. [file 12711_2015_100_MOESM3_ESM.pdf]

Table S2. Accuracy of prediction[1] for traits analyzed[2] with the different relationship matrices[3] implemented in BLUP.

Trait	G^{BASE}	G^{SM}	G^{RR}	G^{BL}	A^{PED}
MY	0.562 (0.028)	0.558 (0.030)	0.567 (0.041)	0.557 (0.023)	0.407 (0.029)
FP	0.548 (0.066)	0.580 (0.058)	0.673 (0.013)	0.678 (0.039)	0.334 (0.047)
PP	0.547 (0.044)	0.565 (0.045)	0.618 (0.033)	0.622 (0.037)	0.351 (0.028)
DC	0.204 (0.020)	0.208 (0.021)	0.219 (0.020)	0.220 (0.021)	0.189 (0.028)
MC	0.123 (0.038)	0.125 (0.037)	0.137 (0.025)	0.129 (0.024)	0.110 (0.035)
BD	0.589 (0.024)	0.595 (0.018)	0.607 (0.009)	0.601 (0.016)	0.483 (0.030)
RW	0.569 (0.053)	0.573 (0.048)	0.592 (0.038)	0.591 (0.040)	0.487 (0.014)
ST	0.625 (0.040)	0.630 (0.034)	0.650 (0.020)	0.649 (0.020)	0.501 (0.025)
SR	0.539 (0.034)	0.545 (0.032)	0.555 (0.022)	0.554 (0.024)	0.404 (0.033)

[1]Accuracy of prediction was measures as the correlation between predicted values and pseude-phenotypes, weighted by the average medoid PTA reliability. The average accuracy and standard deviation across the four medoids is showed.

[2]Traits were milk yield (MY), fat percentage (FP), protein percentage (PP), direct calving ease (DC), maternal calving ease (MC), body depth (BD), rump width (RW), stature (ST), and strength (SR).

Table S3. Gain in accuracy[1] for traits analyzed[2] for the three informed genomic relationship matrices[3] relative to regular GBLUP.

Trait	G^{SM}	G^{RR}	G^{BL}
MY	-0.007	0.009	-0.008
FP	0.058	0.228	0.237
PP	0.034	0.131	0.138
DC	0.021	0.076	0.080
MC	0.019	0.113	0.049
BD	0.011	0.031	0.021
RW	0.007	0.041	0.040
ST	0.009	0.041	0.040
SR	0.011	0.029	0.029

[1]Gain in accuracy of prediction was expressed as the difference in accuracy between the regular genomic matrix and each of the informed genomic matrices, over the accuracy for the latter.

[2]Traits were milk yield (MY), fat percentage (FP), protein percentage (PP), direct calving ease (DC), maternal calving ease (MC), body depth (BD), rump width (RW), stature (ST), and strength (SR).

[3]Relationship matrices were: single marker regression weighted genomic matrix ($\mathbf{G^{SM}}$), Ridge Regression weighted genomic matrix ($\mathbf{G^{RR}}$), Bayesian LASSO weighted genomic matrix ($\mathbf{G^{BL}}$).

Table S4. Bias of prediction[1] for traits analyzed[2] with the different relationship matrices[3] implemented in BLUP.

Trait	G^{BASE}	G^{SM}	G^{RR}	G^{BL}
MY	1.130 (0.181)	1.033 (0.204)	1.029 (0.198)	0.981 (0.096)
FP	1.738 (0.560)	1.476 (0.314)	1.054 (0.132)	1.046 (0.069)
PP	1.479 (0.383)	1.297 (0.243)	1.206 (0.268)	1.134 (0.201)
DC	0.958 (0.393)	0.957 (0.385)	0.901 (0.360)	0.893 (0.358)
MC	0.807 (0.503)	0.803 (0.491)	0.690 (0.316)	0.624 (0.344)
BD	1.305 (0.564)	1.236 (0.458)	1.040 (0.437)	1.170 (0.385)
RW	1.110 (0.305)	1.094 (0.274)	1.063 (0.222)	1.030 (0.197)
ST	1.307 (0.448)	1.225 (0.334)	1.221 (0.327)	1.194 (0.301)
SR	1.195 (0.439)	1.126 (0.369)	1.176 (0.486)	1.143 (0.451)

[1]Bias of prediction was measured as the slope of the linear regression of the predicted values on the weighted pseudo-phenotypes. Values closer to 1 indicate less bias.

[2]Traits were milk yield (MY), fat percentage (FP), protein percentage (PP), direct calving ease (DC), maternal calving ease (MC), body depth (BD), rump width (RW), stature (ST), and strength (SR).

[3]Relationship matrices were: base marker derived genomic matrix ($\mathbf{G}^{\mathbf{BASE}}$), single marker regression weighted genomic matrix ($\mathbf{G}^{\mathbf{SM}}$), Ridge Regression weighted genomic matrix ($\mathbf{G}^{\mathbf{RR}}$), Bayesian LASSO weighted genomic matrix ($\mathbf{G}^{\mathbf{BL}}$).
